# Peptide Mass Spectra from Micrometer-Thick Ice Films Produced with Femtosecond Pulses

**DOI:** 10.1021/acs.analchem.2c01810

**Published:** 2022-09-25

**Authors:** Andrey Krutilin, Sascha W. Epp, Glaynel M. L. Alejo, Frederik Busse, Djordje Gitaric, Hendrik Schikora, Heinrich Schwoerer, Friedjof Tellkamp

**Affiliations:** † Max Planck Institute for the Structure and Dynamics of Matter, Luruper Chaussee 149, Hamburg 22761, Germany

## Abstract

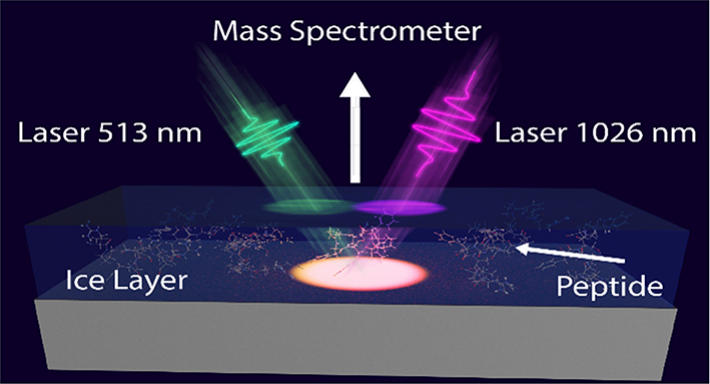

We present a cryogenic mass spectrometry protocol with the capability to detect peptides in the attomole dilution range from ice films. Our approach employs femtosecond laser pulses and implements neither substrate modification nor proton donor agents in the aqueous solution, known to facilitate analyte detection in mass spectrometry. In a systematic study, we investigated the impact of temperature, substrate composition, and irradiation wavelength (513 and 1026 nm) on the bradykinin signal onset. Our findings show that substrate choice and irradiation wavelength have a minor impact on signal intensity once the preparation protocol is optimized. However, if the temperature is increased from −140 to 0 °C, which is accompanied by ice film thinning, a somehow complex picture of analyte desorption and ionization is recognizable, which has not been described in the literature yet. Under cryogenic conditions (−140 °C), obtaining a signal is only possible from isolated sweet spots across the film. If the thin ice film is between −100 and −70 °C of temperature, these sweet spots appear more frequently. Ice sublimation triggered by temperatures above −70 °C leads to an intense and robust signal onset that could be maintained for several hours. In addition to the above findings, we notice that a vibrant fragmentation pattern produced is strikingly similar with both wavelengths. Our findings suggest that while following an optimized protocol, femtosecond mass spectrometry has excellent potential to analyze small organic molecules and peptides with a mass range of up to 2.5 kDa in aqueous solution without any matrix, as employed in matrix-assisted laser desorption/ionization (MALDI) or any substrate surface modification, found in surface-assisted laser desorption/ionization (SALDI).

## Introduction

Matrix-assisted laser desorption/ionization mass spectrometry (MALDI-MS) is the gold standard in analytical chemistry and allows the investigation of large biomolecules due to a soft ionization and desorption process.^1,2^ This method relies on small organic molecules (matrix) to mediate energy deposited from the laser light to the specimen and is critically involved in the desorption as well as the ionization process of the analyte. Due to the complexity of the processes, countless different matrices exist specifically tailored to address the desorption and ionization of a specific group of biomolecules present in the experimental context.^3^ Choosing the suitable candidate is a task that requires frequently the testing of several different matrices on the same specimen.^4,5^ Although MALDI is a widely applied technique, the experimental conditions are strongly acidic, potentially causing precipitation. This may be a reason why some protein signals are of low intensity or absent.

A scenario where mass spectrometry could be performed in situ with minor sample pretreatment under protein-friendly non-denaturing conditions is certainly preferable from a practical as well as scientific point of view. The first approach would be the utilization of water itself as the MALDI matrix. A second approach would be the direct ionization of the constituents without the need for any matrix.

By applying different laser pulse durations and wavelengths, a few studies showed promising results using ice as a matrix.^6−11^ These studies were mainly applying laser pulses longer than a picosecond in duration. Studies with even shorter pulse durations were mainly performed under atmospheric conditions and with the aid of a second process such as electrospray ionization (ESI)^12,13^ producing protein signals for high masses. Nevertheless, studies that worked with samples under vacuum reported severe fragmentation of biological components when utilizing femtosecond pulses on tissue sections.^14^ A noteworthy study by Berry et al. employed a 200 fs pulse length and a 400 nm wavelength light source to give rise to intact proteins of masses of up to 6 kDa.^15^

A technique without the requirement of any matrix is surface-assisted laser desorption/ionization (SALDI),^16,17^ which became popular over the past decade. SALDI utilizes a surface modification, usually on the nanoscale level,^18^ to promote analyte desorption and ionization. The required substrate functionalization can be time-consuming to establish, costly, and is often commercially not available.^19^ Moreover, finding the optimal modification requires testing for each molecule of interest. A special case of SALDI with potential for high-throughput analysis is desorption/ionization on silicon (DIOS), which reduces cost and increases availability.^20^ Most fundamental studies involving SALDI are performed mainly by the aid of nanosecond light sources, whereas other pulse durations are less common.^21−24^

Hitherto, various approaches have been presented in studies of laser desorption under cryogenic conditions,^8,11,15,25^ some only investigating the bulk material and others also observing the effects of layer thickness. The influence of sample preparation, however, has been elusive—mostly due to the effects of condensation from ambient air and the evaporation of the ice layer during evacuation of the sample chamber—and a concise and reproducible protocol for sample preparation was not given.

Although motivated by the work of Berry et al.,^15^ we investigated matrix-free laser desorption mass spectrometry in a very different experimental regime in terms of sample thickness, temperature, and laser pulse energy. To better understand the analyte–laser–substrate interactions, we employ two different laser wavelengths of 1026 and 513 nm. We observed strong vigorous analyte fragmentation, which has not been reported before.^15^ Last but not least, our approach also employs a sample preparation technique capable of producing suitably thin ice films, which are paramount in our experimental framework. By using thin ice films, we can estimate our experimental sensitivity.

## Experimental Section

### Laser System

All experiments were conducted with a Pharos laser system (Light Conversion UAB, Vilnius, Lithuania). The laser emits at a 1026 nm central wavelength with a spectral bandwidth of 5 nm and a temporal full width at half-maximum of 190 fs. Pulse energies and relative standard deviation were frequently measured and on average were below 0.5%. In the experiment, the fundamental (1026 nm) and the second harmonic (513 nm) of the laser is used, while the latter was generated with a half-wave plate (WPH225H, Dayoptics, Fujian, China) and a nonlinear crystal (BBO-654H, Eksma Optics, Vilnius, Lithuania). A harmonic beamsplitter was used to clean the 513 nm from the fundamental (HSY13, ThorLabs, Newton, USA). Both laser beams were coupled into the mass spectrometer at an incidence angle of 35° toward the normal of the sample surface. Focusing was achieved with a single lens (LA1509, *f* = 100 mm, ThorLabs, Newton, USA) for both wavelengths.

### Time-of-Flight Mass Spectrometer

The linear time-of-flight mass spectrometer was designed and constructed in-house. The instrument consists of three main sections: the ion extraction region, the linear flight tube with an incorporated ion beam focusing and steering system, and the detector (respectively from left to right in Figure S1). The flight tube was made out of aluminum pipe sections so that the total length could be varied between 1.06 and 1.14 m. The voltages for the backing plate, extraction plate, and einzel lens were +16, +14.5, and +8.5 kV, respectively. For some experiments, +17 kV was used for the backing plate. All mass spectra were recorded in the positive ion mode.

### Residual Gas Analyzer

The low-pressure rest gas environment in the time-of-flight mass spectrometer was analyzed with a PrismaPlus QME 220 residual gas analyzer (Pfeiffer, Aßlar, Germany).

### Sample Stage

The sample carrier and sample stage were custom-made. The sample carrier was installed onto an electrically insulated translation stage (SLC Series, SmarAct, Oldenburg, Germany), and a connection with a cold finger through ultra-flexible copper braids allowed continuous cooling of the sample except during the transfer into the vacuum.

### Detection

All spectra were acquired with a flange-mounted dual-stage microchannel plate detector assembly (F9890-31 with F1552-011G, Hamamatsu City, Hamamatsu, Japan), which has an effective diameter of 40 mm, a channel diameter of 12 μm, and a gain of 10.^6^ The voltage settings for the front plate, back plate, and anode were −1650, 0, and +50 V, respectively. A 20 dB preamplifier TA1800B with a 1.8 GHz bandwidth (FAST ComTec, Munich, Germany) was used to amplify the signal into a 1 GS/s 8 bit digitizer (Acqiris DC211, Agilent, USA).

### Materials

Bradykinin, angiotensin, and LC–MS/MS water were purchased from Merck (Darmstadt, Germany) and used without further preparation. Peptide mixtures were dissolved in ultrapure water to the desired concentrations and stored at +4 °C before usage. Silicon wafers with two sides polished and *n*-type doping (resistivity 0.1–1.0 ohm·cm^–1^) and a thickness of 300 ± 25 μm were obtained from MicroChemicals GmbH (Ulm, Germany). Indium tin oxide-coated coverslips (resistivity 70–100 Ω, thickness 160 μm) were purchased from SPI Supplies (West Chester, USA). Chalcogenide glass was bought from Vitron (Jena-Maua, Germany).

### Sample Preparation

Thin-film aqueous samples were prepared according to the following protocol: different concentrations of peptides were mixed in pure water. Then, 150–200 nL of the peptide solution was deposited on a silicon wafer, indium tin oxide coverslip, or chalcogenide glass with a size of 18 × 18 mm. After which, a second coverslip was placed on top, causing the sample droplet to spread between the coverslips. This scaffold was then placed above a liquid nitrogen surface for cooling at a convenient rate. The following step is time-critical since the heating block and the coverslip must be simultaneously removed. After roughly 30 s, the upper coverslip was tempered up to 35 °C with a heating block to reduce the adhesion to the ice layer (Figure S2, panels A and B). An extended freezing period can lead to separation failure, and the protocol must be repeated. However, the exact temperature of the sample between the two coverslips cannot be measured. The result is an aqueous thin film exhibiting white light interferences if successfully prepared.

### Transfer Protocol

Figure S3 schematically depicts the sample loading process. The nitrogen used in the experiments had a purity rating of 5.0. This technique retained interference during the loading process (Figure S4), which indicates that the sample thickness is unchanged with respect to the initial sample (panel A). Conversely, Figure S4 (panel B) shows a failed loading process where the sample carrier is covered with ambient air condensate.

### MS Imaging

The sample stage control and data acquisition software was developed in-house (LabVIEW, National Instruments, USA). Two-dimensional images were acquired by moving the stage in steps of 250 μm in the *x*-direction and 100 μm in the *y*-direction. Each pixel’s color intensity correlates with the signal intensity of the bradykinin signal. In total, 441 shots were distributed over an area of roughly 5 × 3 mm, and consecutive runs were recorded by an intermediate 30 μm shift in the *x*-direction, interlacing the different runs of 441 shots for better comparability (Figure S5).

### Microscopy

A commercial bright-field microscope (Axioscope 7, Zeiss Jena, Germany) was used to analyze the substrate surface after laser impact. Images were evaluated using the proprietary software provided by the instrument developer (ZenCore, Version 3.1).

### Data Processing and Availability

Mass spectrometric raw data were recorded with a software provided by Cameca Instruments Inc. (Madison, USA). All data sets were recorded in a single-shot-per-spot (SSpS) mode, and the raw data were further processed with a Python script available on GitHub.^26,27^

## Results and Discussion

### Sample Preparation and Transfer Protocol

A fast and straightforward sample preparation method is crucial to enable routine use of cryogenic mass spectrometry-based techniques. Several studies were carried out under cryogenic conditions, but a detailed protocol for sample preparation was not in scope.^8,11,15,25^ We investigated our sample protocol on silicon, chalcogenide glass (CG), and indium tin oxide (ITO) coverslips. Generally, the sample preparation works with all substrates, but the most reproducible thin films with best homogeneous spreading were achieved with silicon as a substrate, followed by ITO. CG turned out to be fragile with a tendency to fracture. Another drawback shared by ITO and CG is the separation of the pair of top and bottom coverslips that often resulted in an inhomogeneous thin-film layer on the target substrate. Finally, in a shot-to-shot repeatability^28^ experiment, we show that the silicon wafer in conjunction with femtosecond IR-LDI has the highest repeatability rates (Figure S6).

We observed that the most reproducible results were generated when the specimen had the least possible exposure to the ambient air. As a solution the loading arm was flushed with nitrogen (5.0) to minimized the contact between air and the specimen during the loading process Figure S3.

### Cryogenic Stage Characterization

In this study, temperature-dependent measurements were conducted. While the attachment of a Pt1000 sensor to the sample stage made it possible to record a temperature profile, experiments typically requiring high voltage were not simultaneously feasible. However, since the evolution of the measured temperature was highly reproducible, we used the elapsed time to accurately estimate the temperature for a given time point (Figure S7A).

In addition to temperature profile measurement, we also recorded the pressure profile in the mass spectrometer. After the liquid nitrogen supply had been cut, the pressure measurement revealed an elevated pressure level after 70 min (Figure S7B, red trace). Identical measurement without the coverslip and the thin ice film showed no peak at this point (Figure S7B, green trace). Further residual gas analyzer measurements confirmed that water is responsible for the pressure increase in the mass spectrometer environment (Figure S8). Due to this sublimation of water, the water-to-analyte ratio of the film changes at the 65 min mark and with it likely the morphology of the thin film.

### Femtosecond Mass Spectrometry

**Figure 1:**
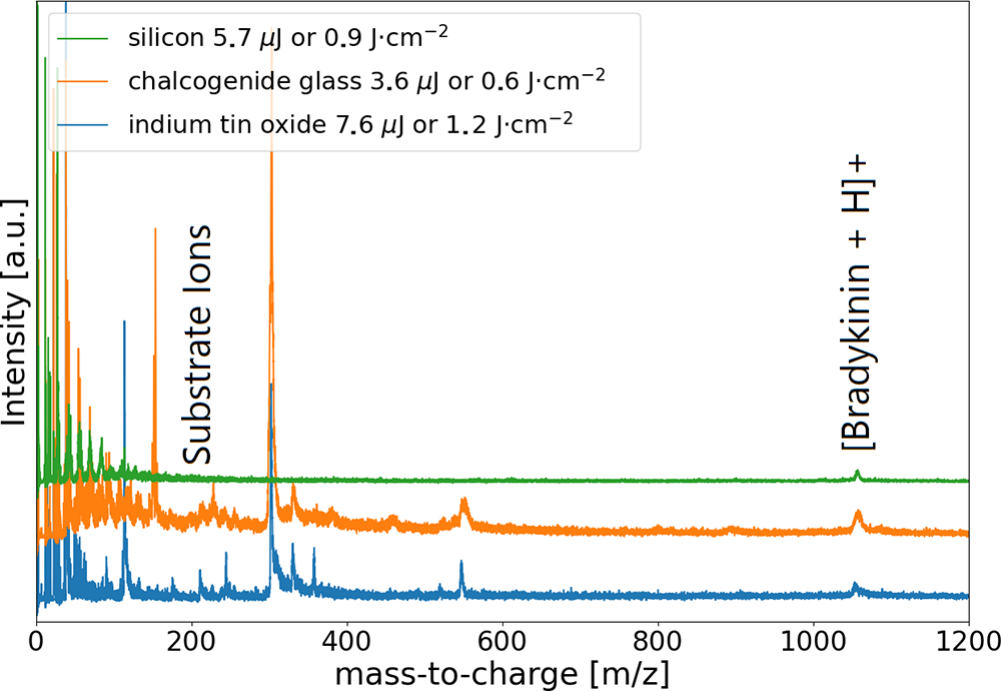
Femtosecond mass spectra of 100 μM bradykinin on three different substrates: chalcogenide glass (orange), silicon (green), and indium tin oxide (blue). For each mass spectrum, 200 nL sample volumes were pipetted. Each mass spectrum is an average of 80–90 single shots. The mass spectra were recorded at −140 °C.

It was possible to obtain a bradykinin signal (1061 *m*/*z*) from all substrates (Figure 1). In addition, well-pronounced substrate signals of up to 550 *m*/*z* were also observed for CG and ITO, whereas for silicon, these signals reached only 150 *m*/*z*. We noticed notable differences in comparison to the hitherto reported results,^15^ because bradykinin signal acquisition was not possible throughout the whole sample area. Instead, there were usually regions that constituted a considerable fraction of the area and provided usable signals and other regions with no signal. Increasing the pulse energies did not generate a sufficient gain for the analyte signal, and instead, the substrate and small mass molecules oversaturated the detector, resulting in an overall reduced analytical performance. Since our mass spectrometer was not equipped with deflection plates or gating rods, suppressing the intensity of low mass ions was limited. Attempts to reduce the low mass signal intensities by reducing the laser pulse energy were unsuccessful due to a direct correlation with analyte signal intensity. The mass spectra with the analyte were collected in an SSpS mode. It was highly reproducible that shots on the same spot did not cause any additional analyte signal, strongly suggesting complete sample desorption with a single shot due to the extremely thin nature of the film. Finally, laser pulse energies used in this work are about sixfold lower than in the aforementioned report.^15^

Furthermore, we compared the bradykinin signal quality on the three different substrates. A convincing signal-to-noise ratio can be obtained on silicon and ITO, while signals on CG suffer from broadening, limiting the reachable resolving power and usability. Since CG is an insulator, the electric field in the extraction region is distorted, leading to low analytical performance. Another drawback of CG, also observable with ITO, is an elevated baseline noise in the mass spectrum. Since other experimental parameters were kept constant, we suspect that this effect is specific to the substrate. The three substrates exhibit different thresholds in laser pulse energies for the bradykinin’s signal onset**.** Differences in the pulse energy might be connected to sample carrier reflectivity for 1026 nm wavelength. The lowest reflectivity values were measured for ITO (0.06), followed by CG (0.41), and then silicon (0.5). To our surprise, we were able to identify an optimal pulse energy with a drop in the signal quality to lower as well as higher pulse energies (Figures S9 and S10). Higher reflectivity increases the energy deposited in the target/analyte by the reflected amount interacting with the analyte a second time and presumably promotes signal onset at lower laser pulse energies.

A very important characteristic in our experiment is the lack of substrate surface pretreatment, as is the case for SALDI or DIOS. The silicon wafers and other coverslips are off-the-shelf products. In addition, we were also able to reuse the coverslips once they were exposed to rigorous cleaning. We like to stress that we do not add any proton donor agents like trifluoroacetic acid used in the previous report^15^ or a particular matrix compound to our water analyte solution. Our protocol points toward an in situ use of aqueous samples with a short preprocessing protocol.

Finally, as mentioned above, cryogenic thin films revealed distinct area “sweet spots” in which signal acquisition was possible. With temperature rise, these areas expanded in dimensions and triggered us to conduct a temperature-dependent study.

### Femtosecond Mass Spectrometry at Elevated Temperatures

By blocking the liquid nitrogen supply, we initiated a temperature rise from −140 °C (Figure S7). For all measurements, laser pulse energy and mass spectrometer settings were unaltered. A mass spectrum was acquired as described in Experimental Section.

**Figure 2:**
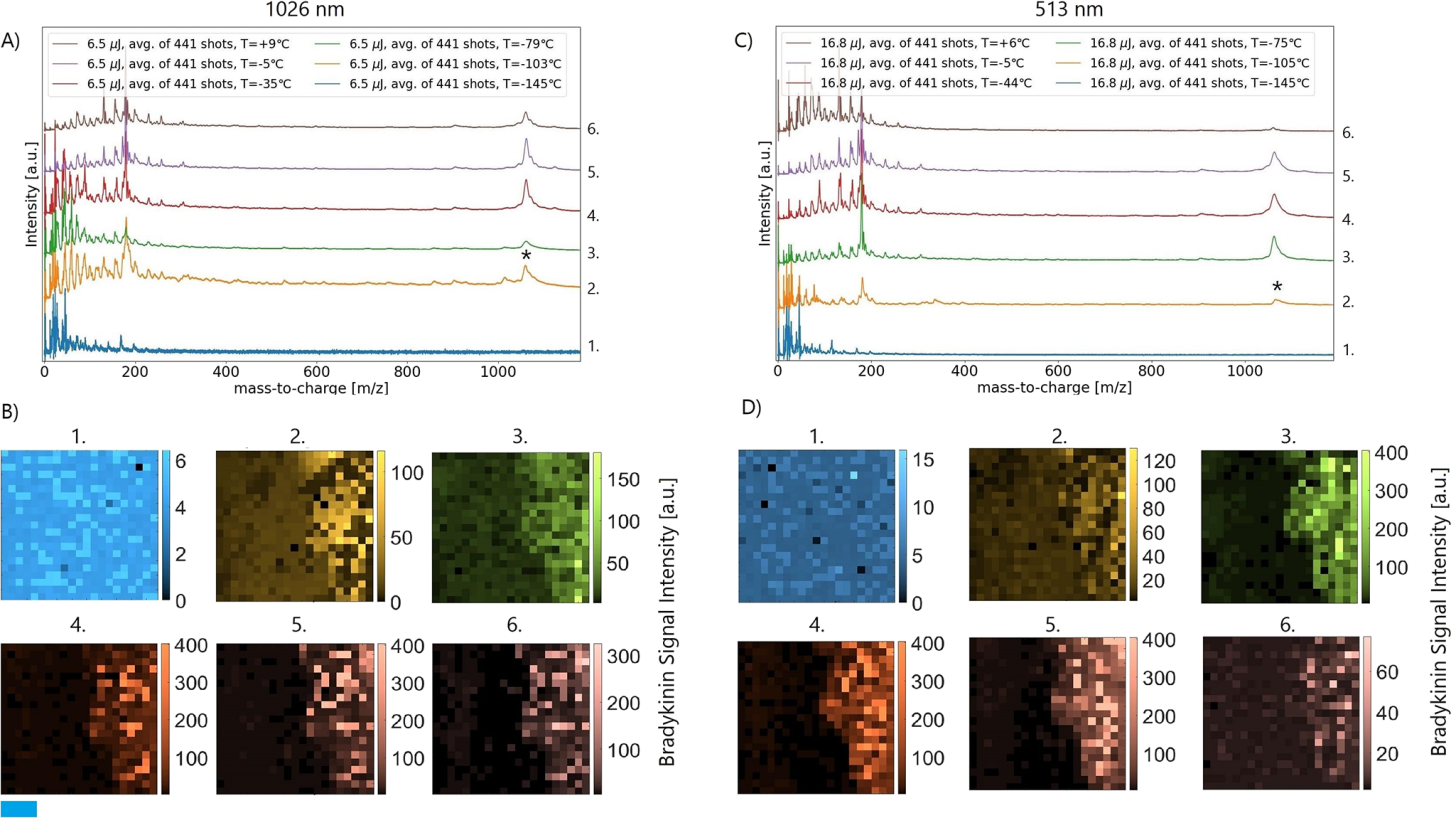
Mass spectra of bradykinin under cryogenic conditions at different temperatures. Overall, 175 nL of a 100 μM analyte solution was pipetted. The asterisk in the figure marks the bradykinin position for all traces. In total, 441 shots were averaged for a single mass spectrum. The color intensity of each pixel correlates with bradykinin signal intensity, which is described by the color bar to the right of an image. The blue box indicates 1 mm. (A) Mass spectra produced by 1026 nm irradiation with an average pulse energy of 6.5 μJ. (B) Corresponding 2D images of bradykinin signal created on an area of 5 × 3 mm (*x* and *y*). (C) Mass spectra produced with 513 nm irradiation with an average pulse energy of 16.8 μJ. (D) Corresponding 2D images of bradykinin signal created on an area of 5 × 3 mm (*x* and *y*).

The mass spectra presented in Figure 2 generally consist of three regions. The origin of the first two ion signal groups between 0 and 300 *m*/*z* as well as the region between 300 and 1000 *m*/*z* will be discussed later. This part will solely focus on the qualitative aspects of the bradykinin signal at different sample stage temperatures.

The blue trace in Figure 2A (panel 1) lacks a bradykinin signal. The corresponding two-dimensional image is shown in Figure 2B (panel 1). This observation might be contradictory to Figure 1, but in this experiment, the mass spectrum was generated by random array shots without searching for sweet spots, giving a more objective picture of signal onset under cryogenic conditions. We verified the result presented here with additional runs. In contrast to the reporting in ref (15), the bradykinin signal was difficult to obtain directly from ice under cryogenic conditions. The discrepancy might be due to the difference in laser pulse energies, sample thickness, or the sample preparation protocol. Attempts to generate a signal with pulse energies of up to 30 μJ were unsuccessful. We cannot exclude the possibility of obtaining a bradykinin signal directly from ice if laser pulse energies are sufficiently increased, but the current setup in our mass spectrometer prevented the realization of such a regime.

In the second recording (Figure 2A, orange trace), the onset of the analyte signal is discernible, with Figure 2B (panel 2) providing the corresponding two-dimensional image. In the last image, an intense signal area on the right of the picture is visible. The accumulation of signal in the right edge of the image was by design as this coverslip exhibits bare silicon on the left side and sample film on the right side. Similar bradykinin signal intensities can be obtained in subsequent runs in a temperature window of −100 and −70 °C or between 50 and 80 min into the warm-up procedure. Since the bradykinin signal is observable, we conclude that two parameters are important for a successful run: first, the water-to-analyte ratio and, second, the film thickness at the current time point. The ideal conditions for both parameters were difficult to obtain in the current setup and required future studies. Furthermore, the experiment also showed that biomolecules can be desorbed and ionized with some residual water.

The third trace or image (Figure 2A, green trace, and Figure 2B, panel 3) was recorded at a temperature of −70 °C when the rest gas pressure peaked, indicating substantial ice sublimation. The second and third mass spectra possess similar signals, although the signal intensity for bradykinin decreased for the latter.

After sublimation, the bradykinin signal intensity is significantly higher (Figure 2A, red trace, and Figure 2B, panel 4). A sharp line between the regions with and without the analyte and bradykinin ions almost saturated the detector. The area with bradykinin signal reveals that the thin film is presumably not homogenous. If this inhomogeneity is with respect to film thickness or analyte ratio remains unknown. The mass spectra of Figure 2B (panels 4, 5, and 6) were recorded in a temperature window between −70 and 0 °C or between 80 and 340 min after initializing the warm-up. Within this temperature and time window, no significant changes in the spectral quality were observed. We did not dedicatedly investigate when the bradykinin signal ultimately faded, but measurement a day later did not produce a signal. Nevertheless, if a similar experiment was repeated with tenfold higher bradykinin concentrations, a signal could be obtained even a day after. To understand the nature of the laser–matter interactions responsible for creating the charged analyte plume, we added a second harmonic wavelength (513 nm) while keeping all other parameters similar to prior measurements. As a result, the second harmonic’s 21 × 21 array is interlaced with the fundamental’s array to assure comparability. The mass spectra produced with the second harmonic and fundamental wavelength had a few minutes delay.

During the experiment, the second harmonic laser pulse energies were twice as high for bradykinin signal onset as for the fundamental wavelength (Figure 2C,D). The reason for the pulse energy difference is the focused area (Figure S11). This discrepancy is introduced through the optical elements in the mass spectrum used for both rays. However, laser pulses at both wavelengths produce the same number of photons per pulse but at different energies per photon. Remarkably, the mass spectra obtained with both wavelengths share the same ions with comparable intensities during and after ice sublimation, independent of whether the second harmonic or the fundamental was employed. Surprisingly, the wavelength plays a subordinate role in desorption and ionization processes in our experimental context. Possible desorption and ionization processes responsible for signal onset are discussed in the section below.

### Fragmentation

Femtosecond pulses interacting with organic materials produce a rich fragmentation pattern in comparison to common activation techniques like collision-induced dissociation or infrared multiphoton dissociation,^29−33^ allowing more complete structural information.^30^ However, these methods were employed on biomolecules in the gas phase. Detection of biomolecule ions and associated fragments from a “neutral” specimen created with femtosecond pulses from a solid substrate has yet to be reported.

Besides substrate signals, more than 15 structurally significant ions originated from the bradykinin molecule on silicon and ITO. Only four ions produced on CG could be annotated as fragments of bradykinin. Two factors may lead to a lower number. First, a severe signal broadening resulted in the overlapping of neighboring peaks. Second, the mass spectra produced on CG tend to generate higher substrate noise, which may be burying low-intensity fragmentation ions. The detailed list of fragmentation ions can be found in Table S1 and Figure S12. Fragmentation is not limited to bradykinin and is also observed with angiotensin (Figure S13). Remarkably, a study performed with bradykinin using a radioactive ionization source reported a very similar distribution as well as intensity of fragments compared to the intact species.^34^ Also, satellite ions are visible in this work, which typically requires higher formation energies for formation.^35,36^ Overall, we observed nonselective bond-breaking throughout the peptide backbone and the existing side-chain groups.^34,37^

### Laser–Substrate Interactions

The results so far (Figure 2) showed that intact bradykinin species, as well as associated fragments, can be observed in the mass spectra during temperature rise. However, high-intensity signals in the lower mass range dominated the mass spectrum.

**Figure 3:**
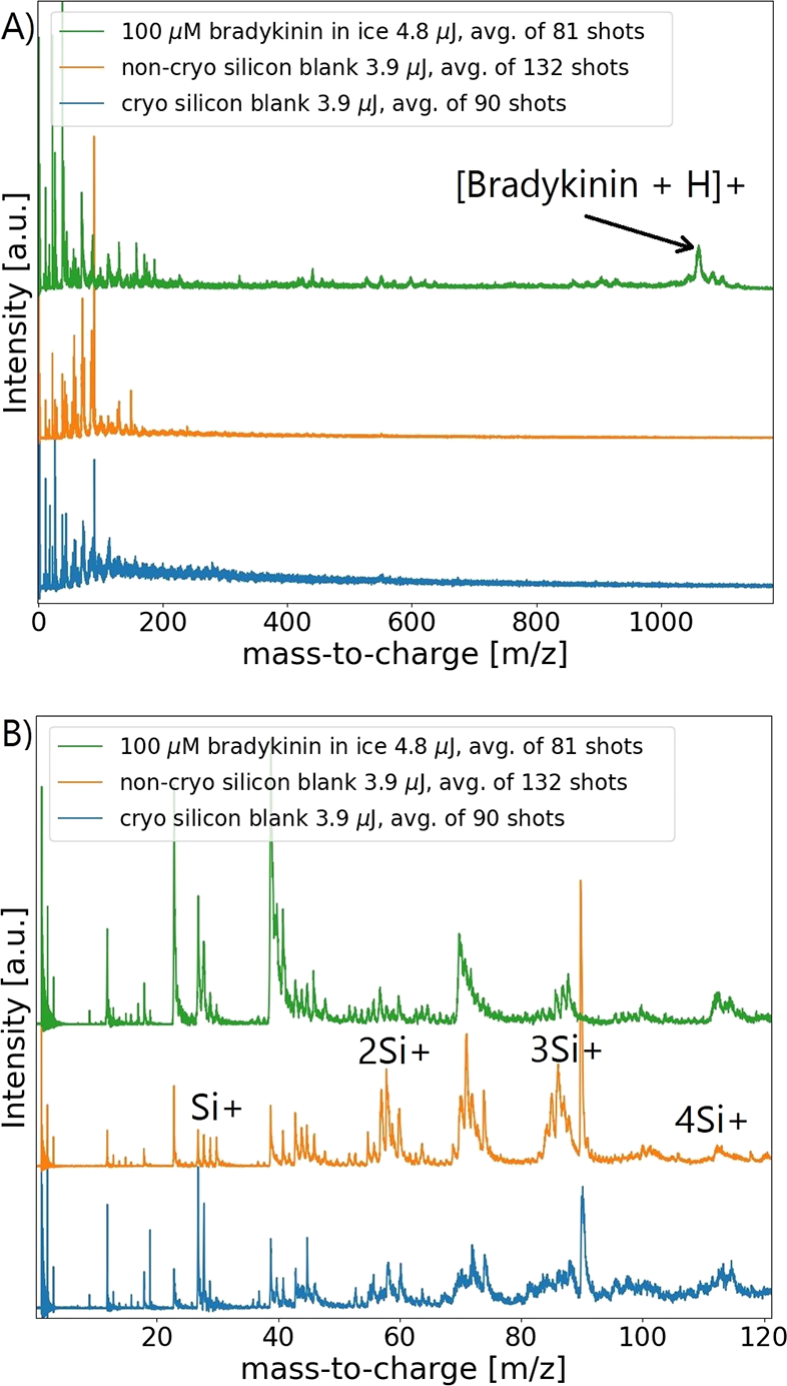
Substrate signals under different conditions produced by femtosecond pulses. (A) Mass spectra of cryogenic (blue) and non-cryogenic (orange) silicon substrates and, for comparison, a mass spectrum of a 100 μM bradykinin thin film (170 nL) on silicon (green). For cryogenic conditions, the sample stage with the silicon substrate was cooled for at least 45 min prior to the measurement. (B) A closer look into the small mass region provides a better visibility. Molecular clusters at similar positions are dominating the mass window for all conditions.

Figure 3 shows the mass spectra of a pure silicon coverslip under cryogenic and non-cryogenic conditions and of an ice film containing 100 μM bradykinin. All three traces show prominent peaks between 0 and 120 *m*/*z*. This observation supports the hypothesis that the signal below 120 *m*/*z* in the experiment related to Figure 2 is dominantly created by the substrate. First, silicon under cryogenic and non-cryogenic conditions exhibits two cluster groups. The first group starts at *m*/*z* = 28, best identified as a singly charged silicon ion, and continues with multimer formation with cluster masses of up to 160 *m*/*z*. Such a cluster formation has already been observed.^38^ The aforementioned cluster groups were also produced when the silicon substrate was covered with a bradykinin ice film (Figure 3, green trace). The latter mass spectrum was recorded during ice sublimation. This observation further solidifies that femtosecond pulses do not solely interact with the analyte solution. Therefore, the laser–substrate interaction needs to be considered in any explanation of the observed effects. The mass spectrum obtained under non-cryogenic conditions produces the highest mass resolution power (Figure 3B, orange trace).

We irradiated the substrate with the laser’s fundamental wavelength while employing different pulse energies **(**Figure S14). The trend is that a double pulse produced a larger crater than a single pulse, while surface modification or damage was almost absent when experimental laser pulse energies were employed (5 μJ). Only at higher fluences, the silicon unveiled crater formation consisting of a melted core with visible ripples^39^ surrounded by a modification zone most probably caused by oxidation/amorphization.^40^

Although damage zones under experimental conditions after laser impact are absent, silicon clusters were observed in the mass spectrum (Figure 3), requiring that substrate ions were released from the surface. A study^40^ suggested that pulses around 1 J·cm^–2^ and 1040 nm, as in our experimental setting, start to remove material. The calculated temperature at this regime was not higher than 1687 K.

### Desorption and Ionization Pathways/Discussion

Femtosecond pulses hitting ice layers with biomolecules produce a dizzying array of phenomena. The interface between the sample and the substrate region is prone to plasma effects above certain intensities, and the electric field distribution is readily inaccessible, and so is the energy of potentially liberated electrons, ions, and clusters, which would be available to initiate desorption and ionization processes. Generally, the possible mixing of different effects will be difficult to disentangle, and further investigations are needed. However, we discuss possible desorption and ionization pathways in the upcoming paragraph.

One of our key observations is that bradykinin signal onset is highly dependent on the sublimation progress. Through thinning of the absorptive ice and water, the substrate is more exposed to the impinging laser light. If more laser light is interacting with the substrate while at the same time the analyte signal is improving, a correlation must be assumed. Further, the substrate irradiation under cryogenic and non-cryogenic conditions with femtosecond pulses forming high-intensity silicon clusters solidifies the laser–substrate interaction (Figure 3).^41^ Although the observations above clearly outline the predominant role of laser–substrate interactions promoting the analyte desorption, we do not find proof that the ejected silicon clusters are mainly responsible for bradykinin desorption. This reasoning is governed by an observation of the bradykinin mass spectra without additional substrate signals (Figure S14). In addition, laser-substrate intertaction can produce a temperature rise, which is another cause for analyte desorption. Laser-induced heat, which is promted laser-substrate interaction is another factor which can drive analyte desorption. Surface melting has been suggested as a potential pathway for ionizing analyte molecules in the SALDI environment.^17,42^ However, in SALDI or DIOS, nanosecond ultraviolet lasers are employed. The interaction between biomolecules and the substrate surface with femtosecond pulses generally differs from that of nanosecond pulses.^43,44^ The heat-affected zone produced after laser pulse impact is proportional to the square root of the pulse duration.^45^ For low-intensity femtosecond pulses, this phenomenon is even more negligible.^46,47^ Our experiment supports that laser-induced heat zones were barely visible under experimental conditions, while only higher pulse energies caused typical heat-induced patterns on the silicon coverslip (Figure S14). Nevertheless, laser-induced temperature rise and the thinning of the ice film might interact beneficially and promote bradykinin desorption.

In addition, for the 1026 nm wavelength, we have found that the analyte signal originates from a fairly narrow 20 μm wide region with local energy densities in excess of 0.6 J·cm^–2^ in the center of the beam (Figure S15B). Within this region, the bradykinin molecule is totally desorbed after the interaction and subsequent pulses on the same region do not produce an analyte signal, although the FWHM diameter of the laser beam is 30 μm and some analyte could interact with the periphery of the laser beam. It is remarkable that the near-IR irradiation and its second harmonic (green) produce such a similar mass spectrometric result, although the boundary conditions are very different concerning beam size, absorption in the substrate, number of photons in the region of 20 μm (where the signal of the near-IR originates), and energy density. Given that the green 513 nm pulses have higher pulse energy, their energy density in the center of the beam is less and so is the number of photons compared to the spatially narrower yet weaker near-IR pulse (Figure S16A).

The desorption of an analyte is the first step toward detection, but in mass spectrometry, typically only ionic species are observable. Due to the limits in the resolution of our mass spectrometer, we cannot make a statement whether the intact bradykinin is arriving at the detector as quasimolecular protonated species, like in MALDI, or with electron deficient or even both. To investigate the possibility of the arrival of an electron-deficient species, an additional experiment with C60 fullerene and toluene was conducted. C60 fullerene and the solvent are neither accepting nor providing protons. However, C60 was detected, hinting that an electron-deficient species cannot be ruled out in our experiment (Figure S17) for bradykinin as our prime molecule of interest. Two possible ionization pathways may be associated with the creation of an electron-deficient species. Either the analyte is ionized through multiphoton ionization^48^ or by electron impact ionization due to liberated electrones based on thermal effects.^49^ Both regimes are plausible and can cause the vibrant fragmentation observed in our experiment (Figure S12).

Multiphoton ionization could create our fragmentation picture through ladder climbing or switching,^48^ but the direct laser–analyte interaction must be assumed here. Therefore, the ionization potentials of experimental constituents must be considered. The ionization potential for silicon, ITO, and water is known,^50−52^ but it is difficult to distinguish for bradykinin. However, peptides with a mass of around 1 kDa have an ionization threshold of around 8 eV.^53^

Another path to desorption and ionization is through a laser-induced plasma. Fluences above 1 J·cm^–2^ are known to cause plasma formation,^54,55^ but in our experiment, we worked at the brink of this threshold, and the impact of a laser-induced plasma is elusive.

However, the briefly discussed phenomena failed to produce an analyte signal when the ice layer was sufficiently thick. In addition, the lack of analyte signal multiple hours later suggests that some volatile components might be crucial for a successful run. Our explanation for the latter observation is the vanishing hydration shell of several water atoms surrounding the analyte. Such a thin layer should stay intact under low-pressure conditions for some hours. This statement is supported by the fact that when the water signal vanished in the mass spectrum, the signal for the analyte vanished some hours later too (Figure 2). Another explanation for the observed phenomena could be a conformational change experienced by the analyte when the water ratio dropped below a critical threshold.

### Analytical Performance

For sample concentration below 10 μM, peptide signal was obtained with a signal-to-noise ratio of 20:1. Assuming a concentration of 10 μM and an ablation crater diameter of 20 μm, the total amount for signal generation adds up to a sensitivity of 25 attomoles, while MALDI imaging reports a sensitivity of about 500 attomoles.^56^^,^^57^ However, in a typical UV-MALDI experiment, peptides are usually detected several magnitudes lower, and the signal is visible for 25 pM solution.^58^

In an additional experiment, we investigated the mass limit and found that the highest obtainable mass is endothelin (2.5 kDa; Figure S18).

## Conclusions

Femtosecond mass spectrometry is a promising candidate to perform real-time analysis on biological specimen without rigorous sample preparation steps. However, femtosecond pulses are known to produce severe fragmentation in biomolecules, reducing the analytical performance. In our study, we investigated frozen aqueous thin films in a temperature-dependent study to provide a better understanding between laser, substrate, and analyte interactions. For study purposes, a sample preparation protocol was developed, leading to an ice film thickness of about 2–3 μm. For all studies, substrate pretreatment common in SALDI and DIOS workflows was not necessary. Peptides embedded in an ice film can be obtained intact under femtosecond laser irradiation (∼1 J·cm^–2^). Different substrates and two wavelengths (513 and 1026 nm) were studied under different temperatures. While silicon as a substrate showed the best results, the two wavelengths employed in this work generated a similar signal intensity. Sample temperature, ultimately affecting the ice thin-film thickness and the water–analyte ratio, has clearly the most significant impact on analyte signal intensity. Three different temperature zones could be identified. Larger thin-film areas exposed to irradiation at temperatures below −100 °C gave unsatisfactory results. However, a bradykinin signal could occasionally be obtained. Samples in the temperature range between −100 and −70 °C generally provide a good signal with a signal-to-noise ratio of 40:1. Following ice sublimation around −70 °C, the signal-to-noise and overall signal quality increased again, and a bradykinin signal could be obtained from a larger area. The sensitivity achieved is around 25 attomoles and is several orders of magnitude lower than previously reported.^15^ In addition, a vibrant fragmentation pattern was produced with both wavelengths (1026 and 513 nm) and led to low-intensity peaks with a similar pattern. From surrounding findings, the role of each ionization pathway producing analyte fragments remains elusive in its quantity. The technique introduced in this work has a great potential in the determination of unknown specimen as sequence coverage is easier to complete if fragments are available.

## Supporting Information Available

Figure S1: CAD drawing of the mass spectrometer and a schematic illustration of the beam guidance into the mass spectrometer. Figure S2: Schematic drawing of the thin-film preparation protocol. Figure S3: Schematic drawing of the home-built loading arm. Figure S4: Bright-field image of the ice thin film during different loading procedures. Figure S5: Illustration of the data acquisition strategy. Figure S6: A histogram of shot-to-shot repeatability for bradykinin signal on three substrates. Figure S7: Temperature and pressure time series in the mass spectrometer. Figure S8: Residual gas analyzer mass spectra at different points after turning off the liquid nitrogen supply. Figure S9: Threshold fluence for bradykinin signal onset on a silicon substrate during 1026 nm irradiation. Figure S10: Threshold fluence for bradykinin signal onset on a silicon substrate during 513 nm irradiation. Figure S11: Laser-induced damage on the silicon coverslip produced with different wavelengths. Figure S12: Femtosecond mass spectrometry producing a vibrant pattern of bradykinin fragment. Figure S13: Angiotensin mass spectrum produced with femtosecond pulses. Figure S14: Bright-field images of craters on a silicon wafer produced with femtosecond pulses. Figure S15: Single-shot mass spectra of 100 μM bradykinin from the same data set. Figure S16: Schematic laser profile of the fundamental and the second harmonic. Figure S17: Temperature-dependent mass spectra of fullerene C60 obtained with 1026 nm irradiation. Figure S18: Mass spectrum of endothelin (PDF)

## Abbreviations

MALDI: matrix-assisted laser desorption/ionization
nL: nanoliter
IR: infrared
CID: collision-induced dissociation
μm: micrometer
kDa: kilodalton
SSpS: single-shot-per-spot
SALDI: surface-assisted laser desorption/ionization
DIOS: desorption/ionization on silicon
μJ: microjoule
μM: micromolar
CG: chalcogenide glass
ITO: indium tin oxide
FWHM: full width at half-maximum

## References

[ref1] Karas M.; Hillenkamp F. Laser Desorption Ionization of Proteins with Molecular Masses Exceeding 10 000 Daltons. Anal. Chem. 1988, 60, 2299–2301. 10.1021/ac00171a028. 3239801

[ref2] Van Belkum A.; Welker M.; Pincus D.; Charrier J.-P.; Girard V. Matrix-Assisted Laser Desorption Ionization Time-of-Flight Mass Spectrometry in Clinical Microbiology: What Are the Current Issues?. Ann. Lab. Med. 2017, 37, 475–483. 10.3343/alm.2017.37.6.475. 28840984 PMC5587819

[ref3] Leopold J.; Popkova Y.; Engel K. M.; Schiller J. Recent Developments of Useful MALDI Matrices for the Mass Spectrometric Characterization of Lipids. Biomolecules 2018, 8, 173 10.3390/biom8040173. PMC6316665 30551655

[ref4] Smolira A.; Wessely-Szponder J. Importance of the Matrix and the Matrix/Sample Ratio in MALDI-TOF-MS Analysis of Cathelicidins Obtained from Porcine Neutrophils. Appl. Biochem. Biotechnol. 2015, 175, 2050–2065. 10.1007/s12010-014-1405-1. 25432341 PMC4322226

[ref5] Steven R. T.; Bunch J. Repeat MALDI MS Imaging of a Single Tissue Section Using Multiple Matrices and Tissue Washes. Anal. Bioanal. Chem. 2013, 405, 4719–4728. 10.1007/s00216-013-6899-9. 23515611

[ref6] Berkenkamp S.; Karas M.; Hillenkamp F. Ice as a Matrix for IR-Matrix-Assisted Laser Desorption/Ionization: Mass Spectra from a Protein Single Crystal. Proc. Natl. Acad. Sci. U. S. A. 1996, 93, 7003–7007. 10.1073/pnas.93.14.7003. 8692933 PMC38924

[ref7] Williams P. Time of Flight Mass Spectrometry of DNA Laser-Ablated from Frozen Aqueous Solutions: Applications to the Human Genome Project. Int. J. Mass Spectrom. Ion Processes 1994, 131, 335–344. 10.1016/0168-1176(93)03887-R.

[ref8] Baltz-Knorr M. L.; Schriver K. E.; Haglund R. F. Infrared Laser Ablation and Ionization of Water Clusters and Biomolecules from Ice. Appl. Surf. Sci. 2002, 197–198, 11–16. 10.1016/S0169-4332(02)00295-7.

[ref9] Nelson R. W.; Thomas R. M.; Williams P. Time-of-flight Mass Spectrometry of Nucleic Acids by Laser Ablation and Ionization from a Frozen Aqueous Matrix. Rapid Commun. Mass Spectrom. 1990, 4, 348–351. 10.1002/rcm.1290040911.

[ref10] Pirkl A.; Soltwisch J.; Draude F.; Dreisewerd K. Infrared Matrix-Assisted Laser Desorption/Ionization Orthogonal-Time-of- Flight Mass Spectrometry Employing a Cooling Stage and Water Ice as a Matrix. Anal. Chem. 2012, 84, 5669–5676. 10.1021/ac300840b. 22670870

[ref11] Witt L.; Pirkl A.; Draude F.; Peter-katalinic J.; Dreisewerd K.; Mormann M.; Peter-Katalinić J.; Dreisewerd K.; Mormann M. Water Ice Is a Soft Matrix for the Structural Characterization of Glycosaminoglycans by Infrared Matrix-Assisted Laser Desorption/Ionization. Anal. Chem. 2014, 86, 6439–6446. 10.1021/ac5008706. 24862464

[ref12] Brady J. J.; Judge E. J.; Levis R. J. Nonresonant Femtosecond Laser Vaporization of Aqueous Protein Preserves Folded Structure. Proc. Natl. Acad. Sci. U. S. A. 2011, 108, 12217–12222. 10.1073/pnas.1105673108. 21746908 PMC3145716

[ref13] Shi F.; Flanigan P. M.; Archer J. J.; Levis R. J. Direct Analysis of Intact Biological Macromolecules by Low-Energy, Fiber-Based Femtosecond Laser Vaporization at 1042 Nm Wavelength with Nanospray Postionization Mass Spectrometry. Anal. Chem. 2015, 87, 3187–3194. 10.1021/ac502563c. 25688836

[ref14] Walker A. V.; Gelb L. D.; Barry G. E.; Subanajouy P.; Poudel A.; Hara M.; Veryovkin I. V.; Bell G. I.; Hanley L. Femtosecond Laser Desorption Ionization Mass Spectrometry Imaging and Multivariate Analysis of Lipids in Pancreatic Tissue. Biointerphases 2018, 13, 03B416 10.1116/1.5016301. PMC5880676 29609468

[ref15] Berry J. I.; Sun S.; Dou Y.; Wucher A.; Winograd N. Laser Desorption and Imaging of Proteins from Ice via UV Femtosecond Laser Pulses. Anal. Chem. 2003, 75, 5146–5151. 10.1021/ac034375p. 14708789

[ref16] Sunner J.; Dratz E.; Chen Y. C. Graphite Surface-Assisted Laser Desorption/Ionization Time-of-Flight Mass Spectrometry of Peptides and Proteins from Liquid Solutions. Anal. Chem. 1995, 67, 4335–4342. 10.1021/ac00119a021. 8633776

[ref17] Müller W. H.; Verdin A.; De Pauw E.; Malherbe C.; Eppe G. Surface-Assisted Laser Desorption/Ionization Mass Spectrometry Imaging: A Review. Mass Spec. Rev. 2022, 1–48. 10.1002/mas.21670 PMC9292874 33174287

[ref18] Picca R. A.; Calvano C. D.; Cioffi N.; Palmisano F. Mechanisms of Nanophase-Induced Desorption in LDI-MS. A Short Review. Nanomaterials 2017, 7, 75 10.3390/nano7040075. PMC5408167 28368330

[ref19] Kim Y. K.; Na H. K.; Kwack S. J.; Ryoo S. R.; Lee Y.; Hong S.; Hong S.; Jeong Y.; Min D. H. Synergistic Effect of Graphene Oxide/MWCNT Films in Laser Desorption/Ionization Mass Spectrometry of Small Molecules and Tissue Imaging. ACS Nano 2011, 5, 4550–4561. 10.1021/nn200245v. 21539346

[ref20] Thomas J. J.; Shen Z.; Crowell J. E.; Finn M. G.; Siuzdak G. Desorption/Ionization on Silicon (DIOS): A Diverse Mass Spectrometry Platform for Protein Characterization. Proc. Natl. Acad. Sci. U. S. A. 2001, 98, 4932–4937. 10.1073/pnas.081069298. 11296246 PMC33141

[ref21] Tang H. W.; Ng K. M.; Lu W.; Che C. M. Ion Desorption Efficiency and Internal Energy Transfer in Carbon-Based Surface-Assisted Laser Desorption/Ionization Mass Spectrometry: Desorption Mechanism(s) and the Design of SALDI Substrates. Anal. Chem. 2009, 81, 4720–4729. 10.1021/ac8026367. 19449861

[ref22] Stolee J. A.; Walker B. N.; Zorba V.; Russo R. E.; Vertes A. Laser-Nanostructure Interactions for Ion Production. Phys. Chem. Chem. Phys. 2012, 14, 8453–8471. 10.1039/c2cp00038e. 22415633

[ref23] Ng K. M.; Chau S. L.; Tang H. W.; Wei X. G.; Lau K. C.; Ye F.; Ng A. M. C. Ion-Desorption Efficiency and Internal-Energy Transfer in Surface-Assisted Laser Desorption/Ionization: More Implication(s) for the Thermal-Driven and Phase-Transition-Driven Desorption Process. J. Phys. Chem. C 2015, 119, 23708–23720. 10.1021/acs.jpcc.5b05957.

[ref24] Lai S. K. M.; Tang H. W.; Lau K. C.; Ng K. M. Nanosecond UV Laser Ablation of Gold Nanoparticles: Enhancement of Ion Desorption by Thermal-Driven Desorption, Vaporization, or Phase Explosion. J. Phys. Chem. C 2016, 120, 20368–20377. 10.1021/acs.jpcc.6b06261.

[ref25] Kraft P.; Alimpiev S.; Dratz E.; Sunner J. Infrared, Surface-Assisted Laser Desorption Ionization Mass Spectrometry on Frozen Aqueous Solutions of Proteins and Peptides Using Suspensions of Organic Solids. J. Am. Soc. Mass Spectrom. 1998, 9, 912–924. 10.1016/S1044-0305(98)00063-4. 9725013

[ref26] Glaynel A. MassSpec Repositoryhttps://github.com/galejo09/massspec.

[ref27] Krutilin A. Femtosecond Mass Spectrometry Repository https://github.com/andrey101010/Femtosecond_Mass_Spectrometry.

[ref28] Brenton A. G.; Godfrey A. R. Accurate Mass Measurement: Terminology and Treatment of Data. J. Am. Soc. Mass Spectrom. 2010, 21, 1821–1835. 10.1016/j.jasms.2010.06.006. 20650651

[ref29] Duffy M. J.; Kelly O.; Calvert C. R.; King R. B.; Belshaw L.; Kelly T. J.; Costello J. T.; Timson D. J.; Bryan W. A.; Kierspel T.; Turcu I. C. E.; Cacho C. M.; Springate E.; Williams I. D.; Greenwood J. B. Fragmentation of Neutral Amino Acids and Small Peptides by Intense, Femtosecond Laser Pulses. J. Am. Soc. Mass Spectrom. 2013, 24, 1366–1375. 10.1007/s13361-013-0653-6. 23817831

[ref30] Kalcic C. L.; Gunaratne T. C.; Jones A. D.; Dantus M.; Reid G. E. Femtosecond Laser-Induced Lonization/Dissociation of Protonated Peptides. J. Am. Chem. Soc. 2009, 131, 940–942. 10.1021/ja8089119. 19128059

[ref31] Raspopov S. A.; El-Faramawy A.; Thomson B. A.; Siu K. W. M. Infrared Multiphoton Dissociation in Quadrupole Time-of-Flight Mass Spectrometry: Top-down Characterization of Proteins. Anal. Chem. 2006, 78, 4572–4577. 10.1021/ac052248i. 16808467

[ref32] Wilson J. S. B. J. J. Infrared Multiphoton Dissociation in Quadropole Ion Traps. Mass Spectrom. Rev. 2009, 28, 390–424. 10.1002/mas.20216. 19294735

[ref33] Crowe M. C.; Brodbelt J. S.; Goolsby B. J.; Hergenrother P. Characterization of Erythromycin Analogs by Collisional Activated Dissociation and Infrared Multiphoton Dissociation in a Quadrupole Ion Trap. J. Am. Soc. Mass Spectrom. 2002, 13, 630–649. 10.1016/S1044-0305(02)00366-5. 12056564

[ref34] Macfarlane R. D. Californium-252 Plasma Desorption Mass Spectrometry. Anal. Chem. 1983, 191, 1247A–1250A. 10.1021/ac00262a002. 4037332

[ref35] Johnson R. S.; Martin S. A.; Biemann K.; Stults J. T.; Watson J. T. Novel Fragmentation Process of Peptides by Collision-Induced Decomposition in a Tandem Mass Spectrometer: Differentiation of Leucine and Isoleucine. Anal. Chem. 1987, 59, 2621–2625. 10.1021/ac00148a019. 3688448

[ref36] Johnson R. S.; Martin S. A.; Biemann K. Collision-Induced Fragmentation of (M + H)+ Ions of Peptides. Side Chain Specific Sequence Ions. Int. J. Mass Spectrom. Ion Processes 1988, 86, 137–154. 10.1016/0168-1176(88)80060-0.

[ref37] Brunet C.; Antoine R.; Dugourd P.; Canon F.; Giuliani A.; Nahon L. Formation and Fragmentation of Radical Peptide Anions: Insights from Vacuum Ultra Violet Spectroscopy. J. Am. Soc. Mass Spectrom. 2012, 23, 274–281. 10.1007/s13361-011-0285-7. 22083590

[ref38] Kato T.; Kobayashi T.; Matsuo Y.; Kurata-Nishimura M.; Oyama R.; Matsumura Y.; Yamamoto H.; Kawai J.; Hayashizaki Y. Comparison between Femtosecond and Nanosecond Laser Ablation of Solution Samples Applied on a Substrate. J. Phys. Conf. Ser. 2007, 59, 372–375. 10.1088/1742-6596/59/1/078.

[ref39] Bonse J.; Baudach S.; Krüger J.; Kautek W.; Lenzner M. Femtosecond Laser Ablation of Silicon-Modification Thresholds and Morphology. Appl. Phys. A: Mater. Sci. Process. 2002, 74, 19–25. 10.1007/s003390100893.

[ref40] Moser R.; Domke M.; Winter J.; Huber H. P.; Marowsky G. Single Pulse Femtosecond Laser Ablation of Silicon-a Comparison between Experimental and Simulated Two-Dimensional Ablation Profiles. Adv. Opt. Technol. 2018, 7, 255–264. 10.1515/aot-2018-0013.

[ref41] Bulgakov A. V.; Ozerov I.; Marine W. Cluster Emission under Femtosecond Laser Ablation of Silicon. Thin Solid Films 2004, 453-454, 557–561. 10.1016/j.tsf.2003.11.136.

[ref42] Werner K.; Gruzdev V.; Talisa N.; Kafka K.; Austin D.; Liebig C. M.; Chowdhury E. Single-Shot Multi-Stage Damage and Ablation of Silicon by Femtosecond Mid-Infrared Laser Pulses. Sci. Rep. 2019, 9, 1–13. 10.1038/s41598-019-56384-0. 31882675 PMC6934619

[ref43] Chichkov B. N.; Momma C.; Nolte S.; von Alvensleben F.; Tünnermann A. Femtosecond, Picosecond and Nanosecond Laser Ablation of Solids. Appl. Phys. A: Mater. Sci. Process. 1996, 63, 109–115. 10.1007/BF01567637.

[ref44] Zhang B.; He M.; Hang W.; Huang B. Minimizing Matrix Effect by Femtosecond Laser Ablation and Ionization in Elemental Determination. Anal. Chem. 2013, 85, 4507–4511. 10.1021/ac400072j. 23607453

[ref45] Zeng X.; Mao X. L.; Greif R.; Russo R. E. Experimental Investigation of Ablation Efficiency and Plasma Expansion during Femtosecond and Nanosecond Laser Ablation of Silicon. Appl. Phys. A: Mater. Sci. Process. 2005, 80, 237–241. 10.1007/s00339-004-2963-9.

[ref46] Leitz K. H.; Redlingshöer B.; Reg Y.; Otto A.; Schmidt M. Metal Ablation with Short and Ultrashort Laser Pulses. Phys. Procedia 2011, 12, 230–238. 10.1016/j.phpro.2011.03.128.

[ref47] Tulej M.; Ligterink N. F.; de Koning C.; Grimaudo V.; Lukmanov R.; Schmidt P. K.; Riedo A.; Wurz P. Current Progress in Femtosecond Laser Ablation/Ionisation Time-of-Flight Mass Spectrometry. Appl. Sci. 2021, 11, 2562 10.3390/app11062562.

[ref48] Ledingham K. W. D.; Singhal R. P. High Intensity Laser Mass Spectrometry — a Review. Int. J. Mass Spectrom. Ion Processes 1997, 163, 149–168. 10.1016/S0168-1176(97)00015-3.

[ref49] Petrov G. M.; Davidson A.; Gordon D.; Hafizi B.; Peñano J. Thermionic Emission of Electrons from Metal Surfaces in the Warm Dense Matter Regime. Phys. Plasmas 2021, 28, 083503 10.1063/5.0054955.

[ref50] Kostko O.; Leone S. R.; Duncan M. A.; Ahmed M. Determination of Ionization Energies of Small Silicon Clusters with Vacuum Ultraviolet Radiation. J. Phys. Chem. A 2010, 114, 3176–3181. 10.1021/jp9091688. 20017531

[ref51] Snow K. B.; Thomas T. F. Mass Spectrum, Ionization Potential, and Appearance Potentials for Fragment Ions of Sulfuric Acid Vapor. Int. J. Mass Spectrom. Ion Processes 1990, 96, 49–68. 10.1016/0168-1176(90)80041-Z.

[ref52] Fukano T.; Motohiro T.; Ida T.; Hashizume H. Ionization Potentials of Transparent Conductive Indium Tin Oxide Films Covered with a Single Layer of Fluorine-Doped Tin Oxide Nanoparticles Grown by Spray Pyrolysis Deposition. J. Appl. Phys. 2005, 97, 084314 10.1063/1.1866488.

[ref53] Cui W.; Thompson M. S.; Reilly J. P. Pathways of Peptide Ion Fragmentation Induced by Vacuum Ultraviolet Light. J. Am. Soc. Mass Spectrom. 2005, 16, 1384–1398. 10.1016/j.jasms.2005.03.050. 15979330

[ref54] Hada M.; Zhang D.; Pichugin K.; Hirscht J.; Kochman M. A.; Hayes S. A.; Manz S.; Gengler R. Y. N.; Wann D. A.; Seki T.; Moriena G.; Morrison C. A.; Matsuo J.; Sciaini G.; Miller R. J. D. Cold Ablation Driven by Localized Forces in Alkali Halides. Nat. Commun. 2014, 5, 1–8. 10.1038/ncomms4863 24835317

[ref55] Cavalleri A.; Sokolowski-Tinten K.; Bialkowski J.; Schreiner M.; von der Linde D. Femtosecond Melting and Ablation of Semiconductors Studied with Time of Flight Mass Spectroscopy. J. Appl. Phys. 1999, 85, 3301–3309. 10.1063/1.369675.

[ref56] Chughtai K.; Heeren R. M. A. Mass Spectrometric Imaging for Biomedical Tissue Analysis. Chem. Rev. 2010, 110, 3237–3277. 10.1021/cr100012c. 20423155 PMC2907483

[ref57] Norris J. L.; Caprioli R. M. Analysis of Tissue Specimens by Matrix-Assisted Laser Desorption/Ionization Imaging Mass Spectrometry in Biological and Clinical Research. Chem. Rev. 2013, 113, 2309–2342. 10.1021/cr3004295. 23394164 PMC3624074

[ref58] Musharraf S. G.; Bibi A.; Shahid N.; Najam-ul-Haq M.; Ambreen N.; Khan M.; Khan K. M.; Choudhary M. I.; Rahman A. U. Benzimidazole, Coumrindione and Flavone Derivatives as Alternate UV Laser Desorption Ionization (LDI) Matrices for Peptides Analysis. Chem. Cent. J. 2013, 7, 1–13. 10.1186/1752-153X-7-77. 23621998 PMC3680071

