# Supplementary material for: Peptide Mass Spectra from Micrometer-Thick Ice Films Produced with Femtosecond Pulses
Source: Anal Chem. 2022 Sep 25;94(39):13359–67. doi: 10.1021/acs.analchem.2c01810 (PMC9535622; doi:10.1021/acs.analchem.2c01810)
Supplement: Supplementary file 1 — ac2c01810_si_001.pdf [file ac2c01810_si_001.pdf]

Supporting Information

Peptide Mass Spectra from Micrometer Thick Ice Films Produced with Femtosecond Pulses

Andrey Krutilin†*, Sascha W. Epp†, Glaynel M. L. Alejo~, Frederik Busse†, Djordje Gitaric†, Hendrik Schikora†, Heinrich Schwoerer†, and Friedjof Tellkamp†

† Max Planck Institute for the Structure and Dynamics of Matter, Luruper Chaussee 149, 22761 Hamburg, Germany

~ Department of Physics & Astronomy, University of British Columbia, Vancouver, BC V6T 1Z1, Canada

*Corresponding Author: Andrey Krutilin: Email Andrey.krutilin@mpsd.mpg.de

Contents

Figure S 1 Linear time-of-flight mass spectrometer. A) A CAD drawing from above and without power supplies. B) Schematic drawing of the instrument. The blue line indicates the path of the laser beam into the instrument. 5

Figure S 2 Sample preparation workflow. A) An illustration of sample preparation steps B) Heating block applied for the removal of the upper coverslip C) A thin film with pronounced white light interferences. Our sample preparation protocol consumed 175 to 200 nanoliters, while the spreading area is estimated to be about 0.64 cm^2, which leads to a mean film thickness of approximately 3 µm. 6

Figure S 3 Sample transfer system to minimize ambient air condensation. A) Prior to the attachment of the sample stage to the loading arm, the case is flooded with nitrogen 5.0. B) After loading, the valve for nitrogen and the sample inlet are closed. Then, the valve for the scroll pump and the turbo pump is opened and the depressurization begins. Finally, the loading arm is attached to the mass spectrometer and the sample carrier is mounted...6

Figure S 4 Bright field images inside the mass spectrometer. A) The image shows a thin film with interferences when loading was successful. Here the visible interference pattern stays intact, which indicates that the sample thickness is also unchanged concerning the initial sample preparation B) Failed sample loading procedure resulting in a white layer of condensed ambient air. Conversely, panel B shows a failed loading process where the sample carrier is covered with ambient air condensate, which ultimately changes the surface and the thickness of the specimen and requires the repetition of the sample preparation protocol. 7

Figure S 5 Data acquisition strategy to minimize biases introduced with sample inhomogeneity. A square represents a single laser shot on a substrate. A set of the same color squares represents a group of laser shots to acquire a single mass spectrum. The color squares mark the area where the second harmonic (513 nm) of the laser was employed, while the blurred dark squares represent the pattern for data acquisition with the fundamental (1026 nm) wavelength. The data acquisition for the fundamental is the same as for the second harmonic. Overlapping of consecutive laser shots was avoided by proper distancing (see figure). In total, for a single mass spectrum, a 21 x 21 shot array was scanned resulting in 441 shot for a single mass spectrum. 8

Figure S 6 A histogram of shot-to-shot repeatability for bradykinin signal on three substrates. 51 datasets were acquired by irradiating a thin film of 100 µM bradykinin with 1026 nm femtosecond laser pulses. Each dataset contains 300-350 single-shot spectra acquired at a sample stage between -95 °C and -70 °C. The distribution of datasets with various shot-to-shot repeatabilities is skewed to the left. Half of the data have 70 % - 90 % shot-to-shot repeatability, with a median of 70 %. For each dataset, shot-to-shot repeatability was calculated and plotted into the histogram. A successful mass spectrum is considered when the signal-to-noise ratio is 3:1 or higher. The colors represent the following substrates: Silicon (green), Indium tin oxide (blue), and chalcogenide glass (orange)............9

Figure S 7 Temperature and pressure time series in the mass spectrometer. A) For the first 30 minutes before t = 0 min., the sample stage is cooled, and the temperature drops. After t = 0 the temperature gradually increases. B) The green and red traces show the pressure profile with a coverslip and sample and an empty sample carrier, respectively. In the beginning, the pressure increased due to micro-leaks in the liquid nitrogen pipeline. 10

Figure S 8 Residual gas analyzer mass spectra at different points after turning off the liquid nitrogen supply. The experiment started with a sample stage at -140 ℃ . A-F) Electronic ionization mass spectra with a mass range from 0 to 100 m/z. The asterisks marks the water radical ion position in the mass

spectrum. G) Pressure profile in the mass spectrometer during the experiment. The color bars in the graph indicate when a mass spectrum was recorded and match the colors in A-F). At the beginning of the experiment, the first mass spectrum lacks a water ion signal. When the pressure starts to increase, the water ion signal also surges in the mass spectrum. At the highest pressure point inside the mass spectrometer, the water ion signal is also high. Only after the pressure stabilizes the water ion signal also decreases. The unit for the y-axis of the EI mass spectra is 10^{-10} A and for the pressure profile graph 10^{-6} mbar. 11

Figure S 9 Threshold fluence for bradykinin signal onset on a silicon substrate during 1026 nm irradiation. During the investigation, the temperature at the sample stage was estimated to – 70 ºC. While pulse energies between 2.8 and 3.2 µJ missed producing an analyte signal, higher pulse energies between 3.8 and 4.4 µJ gave a signal with a high signal-to-noise ratio. Pulse energies above 5 µJ did not produce a signal. A possible explanation is the intense substrate signals (not in the image), which oversaturates the detector and reduces analytical performance. For the experiment 100 µM bradykinin solution and 170 nanoliters were used. 12

Figure S 10 Threshold fluence for bradykinin signal onset on a silicon substrate during 513 nm irradiation. During the investigation, the temperature at the sample stage was estimated to – 70 ºC. While pulse energies below 11 µJ missed producing an analyte signal, higher pulse energies between 13.6 and 16.5 µJ gave a signal with a high signal-to-noise ratio. Pulse energies above 19.4 µJ did not produce a signal. A possible explanation is the intense substrate signals (not in the image), which oversaturates the detector and reduces analytical performance. For the experiment 100 µM bradykinin solution and 170 nanoliters were used. 12

Figure S 11 Laser-induced damage on the silicon coverslip produced with different wavelengths. A) Single shots per spot with an average pulse energy of 30 µJ were used to produce the craters with the second harmonic wavelength. B) Single shots per spot with an average pulse energy of 20 µJ were used to produce the craters with the fundamental wavelength. 13

Figure S 12 Femtosecond mass spectrometry produces a vibrant pattern of bradykinin fragments. A) Mass spectrum of 100 µM bradykinin on silicon. B) Dotted lines indicate a possible Proline cleavage for the creation of c_n and z_n ions. An interesting observation was made with the amino acid Proline. Its position in bradykinin is at second, third, and seventh place. Fragment ions of the series c_n and z_n were missing at these positions. Since these fragments evolve through the cleavage between secondary nitrogen and alpha-carbon, the amino acid's structure must be considered. Proline possesses a five-member ring, which is included in the peptide backbone. The absence of fragment peaks suggests the unlikeliness of the double-cleavage event. For the same reasoning, z_3, z_7, and z_8 C-terminal fragments were not detected. 14

Figure S 13 Angiotensin mass spectrum produced with femtosecond pulses. The wavelength was 1026 nm and the specimen concentration 100 µM. In the mass range between 0 and 300 m/z substation ion signals are dominating. Smaller peaks between 300 and 1300 m/z are associated with angiotensin fragments. 15

Figure S 14 Brightfield images of craters on a silicon wafer produced with femtosecond pulses. The irradiation wavelength was 1026 nm. The craters on the top row of the image were produced by a single shot on the same spot, while in the bottom row the craters were produced by a double shot. The experiment was conducted during ice sublimation to mimic the experimental conditions. The white bar indicates 100 µm. 15

Figure S 15 Single shot mass spectra of 100 µM bradykinin from the same data set. A) Bradykinin arrives at the detector almost as a single species. B) Bradykinin signal is accompanied by substrate ions. 16

Figure S 16 Laser profile of the second harmonic (513 nm) and fundamental (1026 nm) during ablation. A) A comparison between 513 nm (green) and 1026 nm (red). B) Bradykinin signal onset dependence on the distance of adjacent pulses. 16

Figure S 17 Temperature-dependent mass spectra of fullerene C60 obtained with 1026 nm irradiation. The analyte concentration was 1 mM, and the pulse energies were between 13 – 15 µJ. A) A full range mass spectrum. B) A zoom-in of the original mass spectrum in the range between 700 – 2700 m/z. .17

Figure S 18 Mass limit detection. Endothelin (2,491 kDa, 100 µM) is the largest detected molecule in the present work. 17

Table S 1 Fragmentation ions from bradykinin observed on different substrates. The masses presented in this table include a proton attachment. Indium tin oxide and chalcogenide glass are abbreviated as ITO and CG, respectively. 18

A)

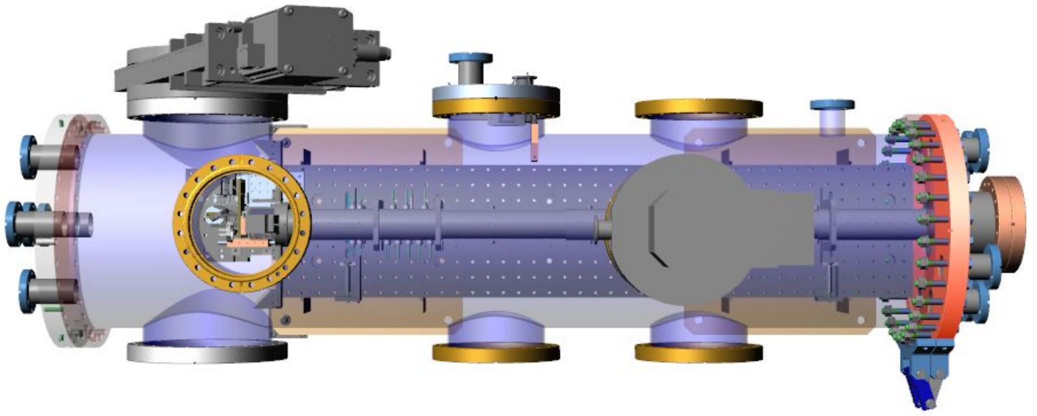

B)

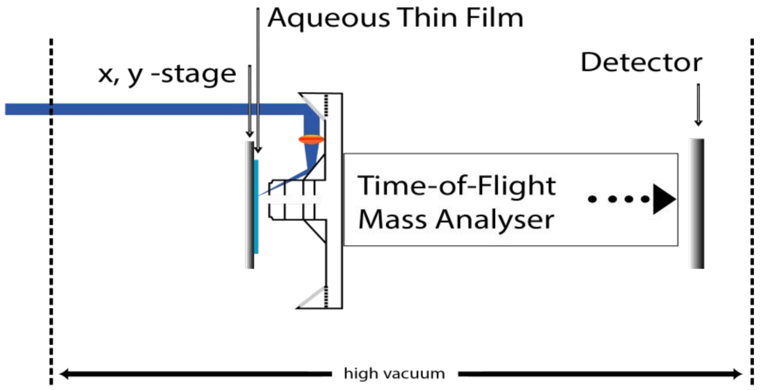

Figure S 1 Linear time-of-flight mass spectrometer. A) A CAD drawing from above and without power supplies. B) Schematic drawing of the instrument. The blue line indicates the path of the laser beam into the instrument.

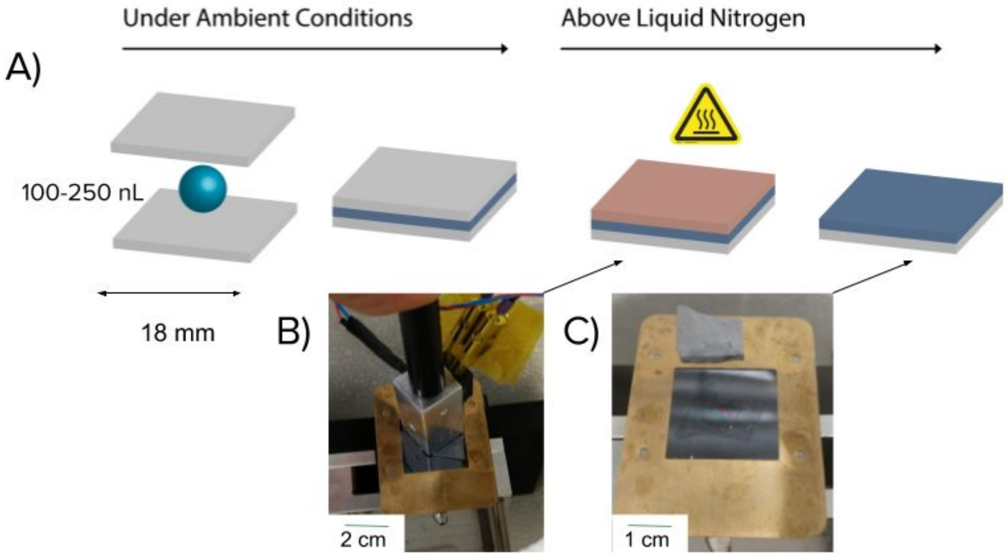

Figure S 2 Sample preparation workflow. A) An illustration of sample preparation steps B) Heating block applied for the removal of the upper coverslip C) A thin film with pronounced white light interferences. Our sample preparation protocol consumed 175 to 200 nanoliters, while the spreading area is estimated to be about 0.64 cm^2, which leads to a mean film thickness of approximately 3 µm.

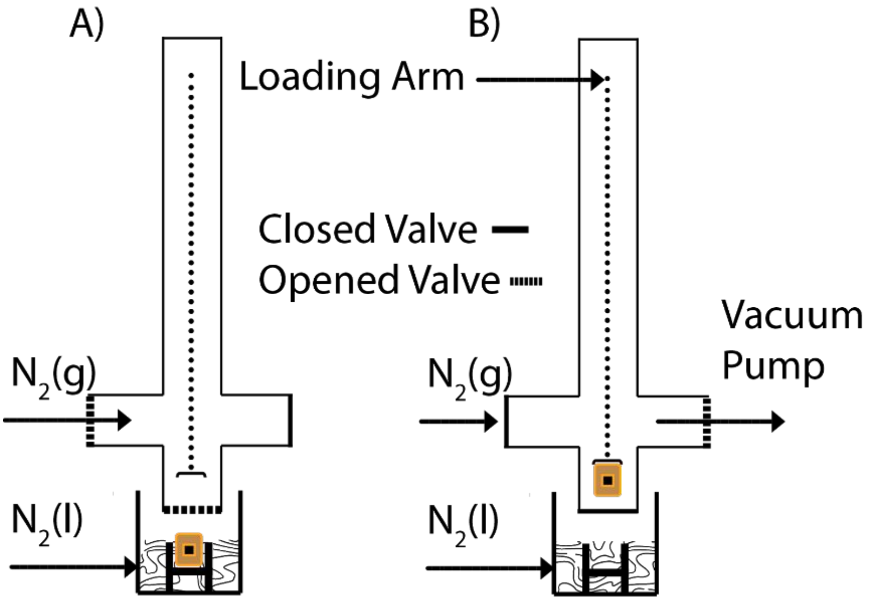

Figure S 3 Sample transfer system to minimize ambient air condensation. A) Prior to the attachment of the sample stage to the loading arm, the case is flooded with nitrogen 5.0. B) After loading, the valve for nitrogen and the sample inlet are closed. Then, the valve for the scroll pump and the turbo pump is opened and the depressurization begins. Finally, the loading arm is attached to the mass spectrometer and the sample carrier is mounted.

A)

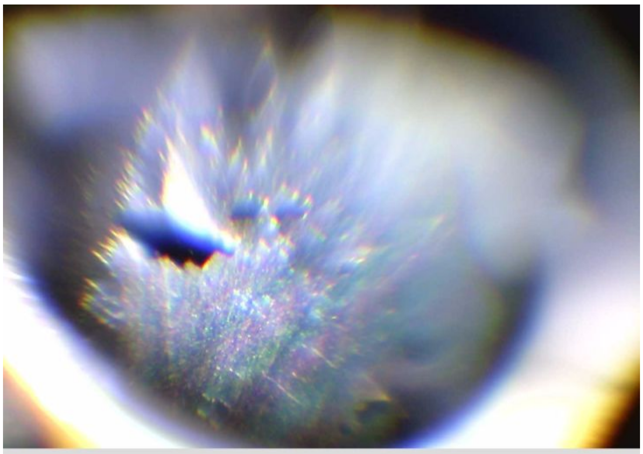

B)

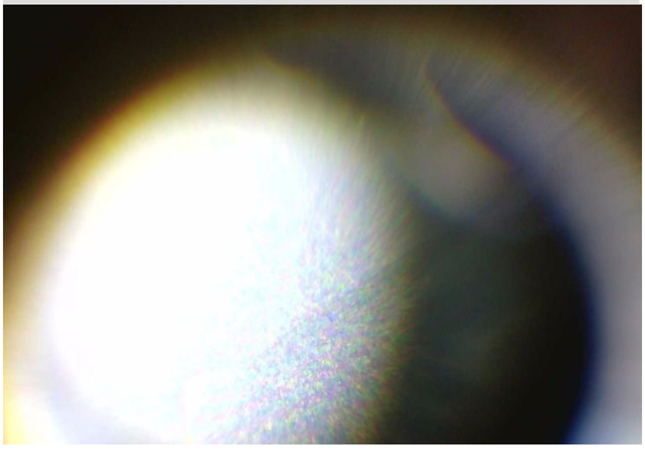

Figure S 4 Bright field images inside the mass spectrometer. A) The image shows a thin film with interferences when loading was successful. Here the visible interference pattern stays intact, which indicates that the sample thickness is also unchanged concerning the initial sample preparation B) Failed sample loading procedure resulting in a white layer of condensed ambient air. Conversely, panel B shows a failed loading process where the sample carrier is covered with ambient air condensate, which ultimately changes the surface and the thickness of the specimen and requires the repetition of the sample preparation protocol.

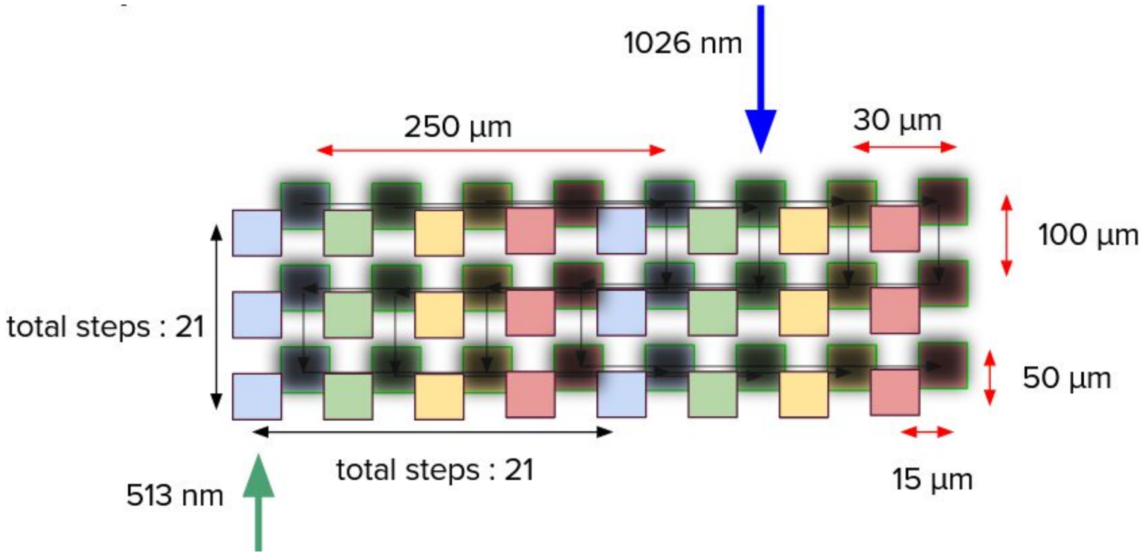

Figure S 5 Data acquisition strategy to minimize biases introduced with sample inhomogeneity. A square represents a single laser shot on a substrate. A set of the same color squares represents a group of laser shots to acquire a single mass spectrum. The color squares mark the area where the second harmonic (513 nm) of the laser was employed, while the blurred dark squares represent the pattern for data acquisition with the fundamental (1026 nm) wavelength. The data acquisition for the fundamental is the same as for the second harmonic. Overlapping of consecutive laser shots was avoided by proper distancing (see figure). In total, for a single mass spectrum, a 21 x 21 shot array was scanned resulting in 441 shot for a single mass spectrum.

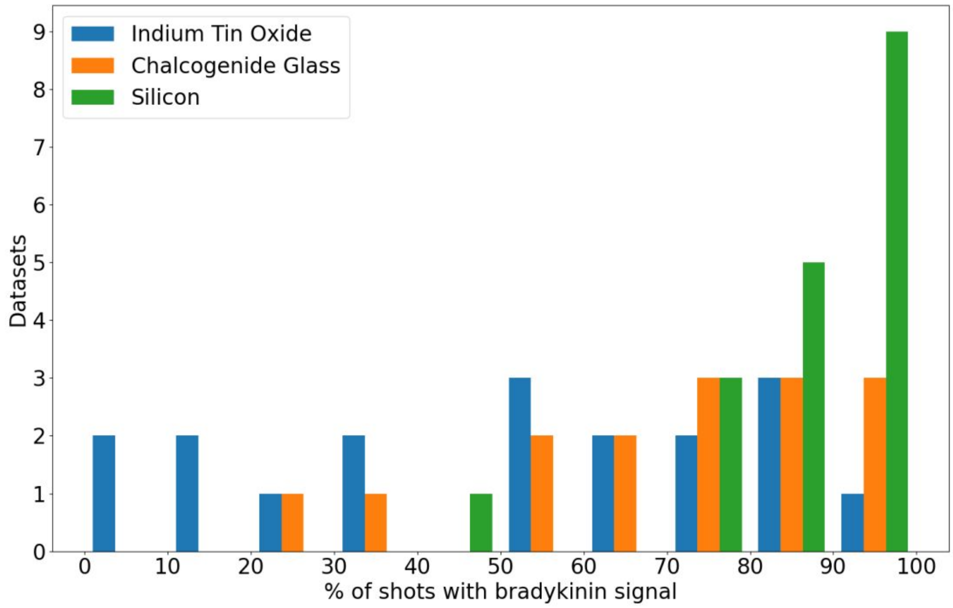

Figure S 6 A histogram of shot-to-shot repeatability for bradykinin signal on three substrates. 51 datasets were acquired by irradiating a thin film of 100 µM bradykinin with 1026 nm femtosecond laser pulses. Each dataset contains 300-350 single-shot spectra acquired at a sample stage between -95 °C and -70 °C. The distribution of datasets with various shot-to-shot repeatabilities is skewed to the left. Half of the data have 70 % - 90 % shot-to-shot repeatability, with a median of 70 %. For each dataset, shot-to-shot repeatability was calculated and plotted into the histogram. A successful mass spectrum is considered when the signal-to-noise ratio is 3:1 or higher. The colors represent the following substrates: Silicon (green), Indium tin oxide (blue), and chalcogenide glass (orange).

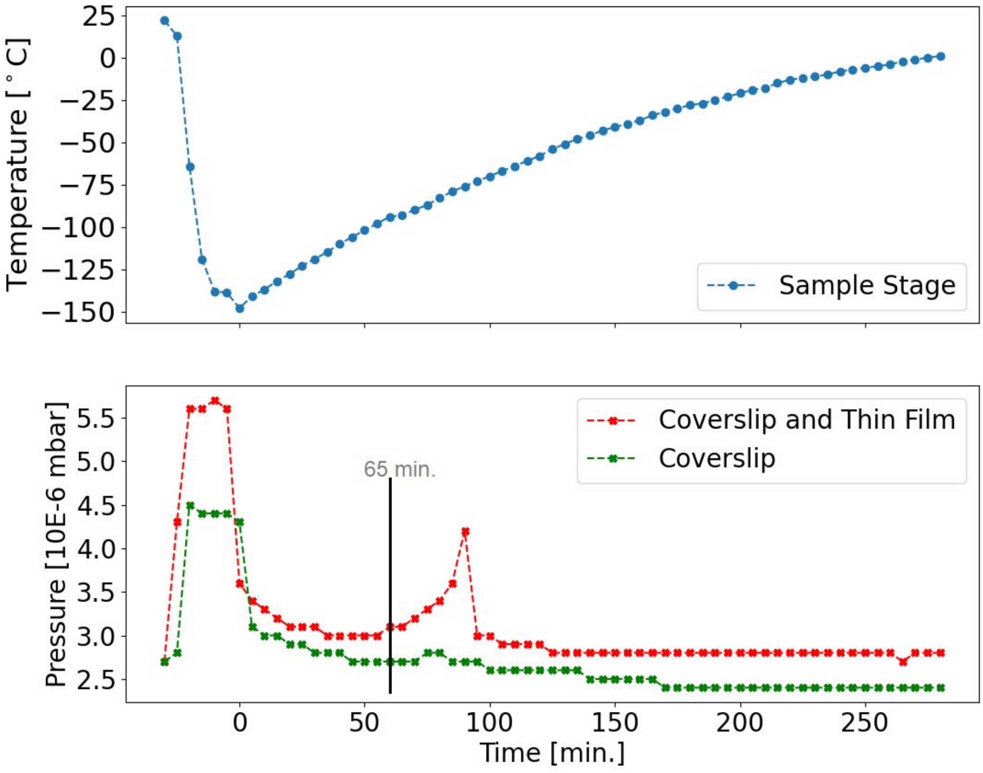

Figure S 7 Temperature and pressure time series in the mass spectrometer. A) For the first 30 minutes before t = 0 min., the sample stage is cooled, and the temperature drops. After t = 0 the temperature gradually increases. B) The green and red traces show the pressure profile with a coverslip and sample and an empty sample carrier, respectively. In the beginning, the pressure increased due to micro-leaks in the liquid nitrogen pipeline.

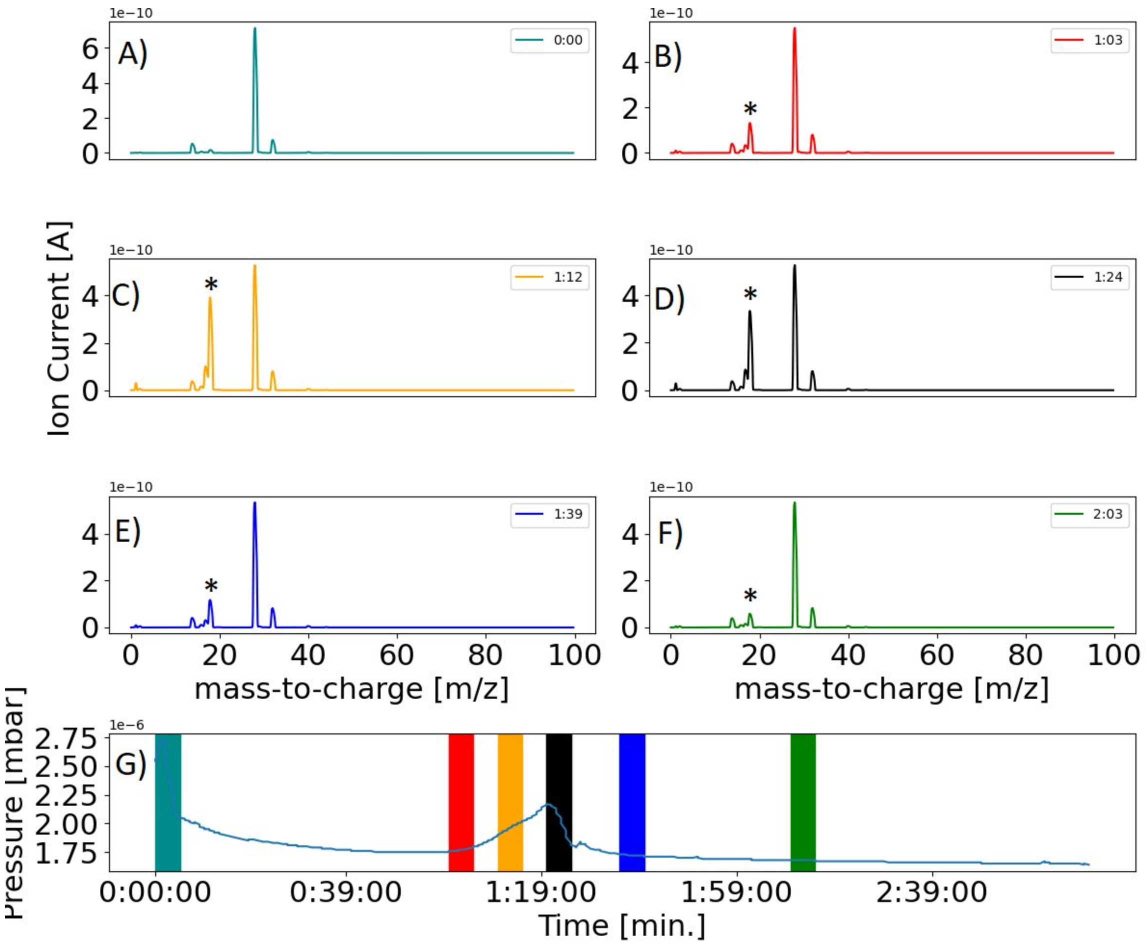

Figure S 8 Residual gas analyzer mass spectra at different points after turning off the liquid nitrogen supply. The experiment started with a sample stage at -140 ℃ . A-F) Electronic ionization mass spectra with a mass range from 0 to 100 m/z. The asterisks marks the water radical ion position in the mass spectrum. G) Pressure profile in the mass spectrometer during the experiment. The color bars in the graph indicate when a mass spectrum was recorded and match the colors in A-F). At the beginning of the experiment, the first mass spectrum lacks a water ion signal. When the pressure starts to increase, the water ion signal also surges in the mass spectrum. At the highest pressure point inside the mass spectrometer, the water ion signal is also high. Only after the pressure stabilizes the water ion signal also decreases. The unit for the y-axis of the EI mass spectra is 10^{-10} A and for the pressure profile graph 10^{-6} mbar.

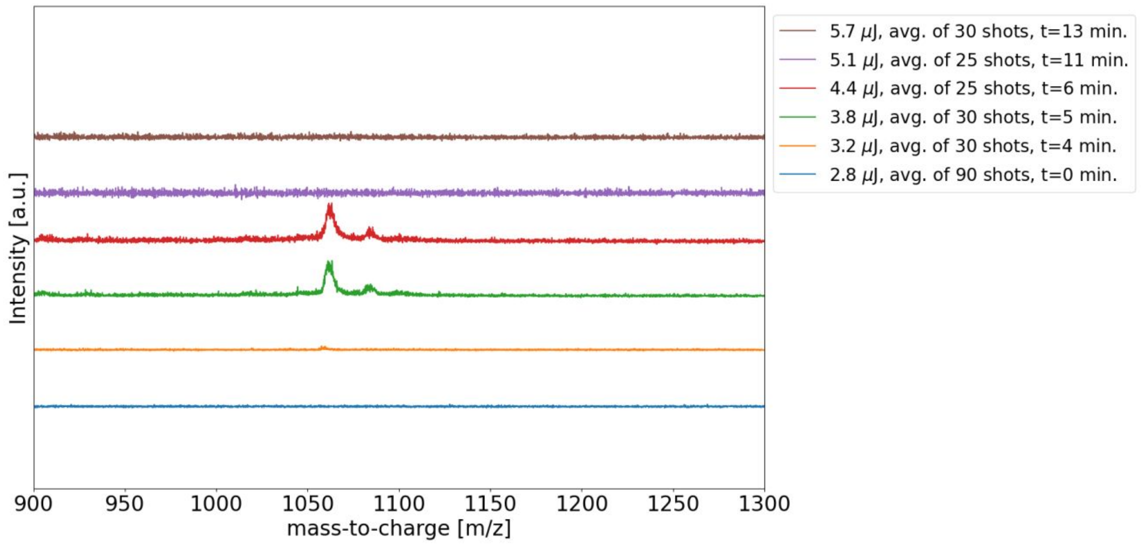

Figure S 9 Threshold fluence for bradykinin signal onset on a silicon substrate during 1026 nm irradiation. During the investigation, the temperature at the sample stage was estimated to – 70 °C. While pulse energies between 2.8 and 3.2 µJ missed producing an analyte signal, higher pulse energies between 3.8 and 4.4 µJ gave a signal with a high signal-to-noise ratio. Pulse energies above 5 µJ did not produce a signal. A possible explanation is the intense substrate signals (not in the image), which oversaturates the detector and reduces analytical performance. For the experiment 100 µM bradykinin solution and 170 nanoliters were used.

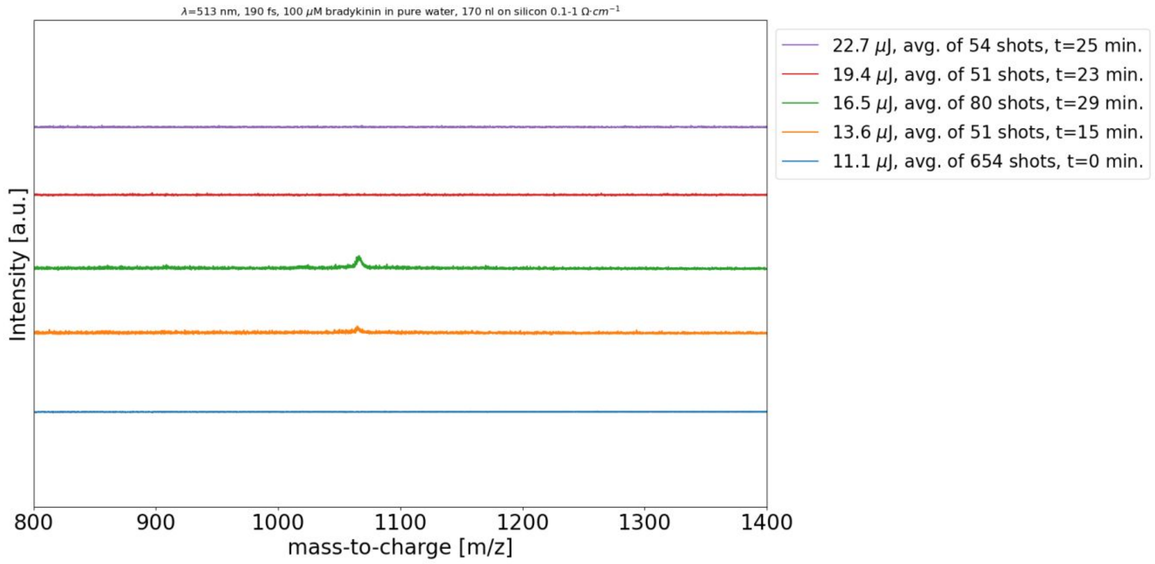

Figure S 10 Threshold fluence for bradykinin signal onset on a silicon substrate during 513 nm irradiation. During the investigation, the temperature at the sample stage was estimated to – 70 °C. While pulse energies below 11 µJ missed producing an analyte signal, higher pulse energies between 13.6 and 16.5 µJ gave a signal with a high signal-to-noise ratio. Pulse energies above 19.4 µJ did not produce a signal. A possible explanation is the intense substrate signals (not in the image), which oversaturates the detector and reduces analytical performance. For the experiment 100 µM bradykinin solution and 170 nanoliters were used.

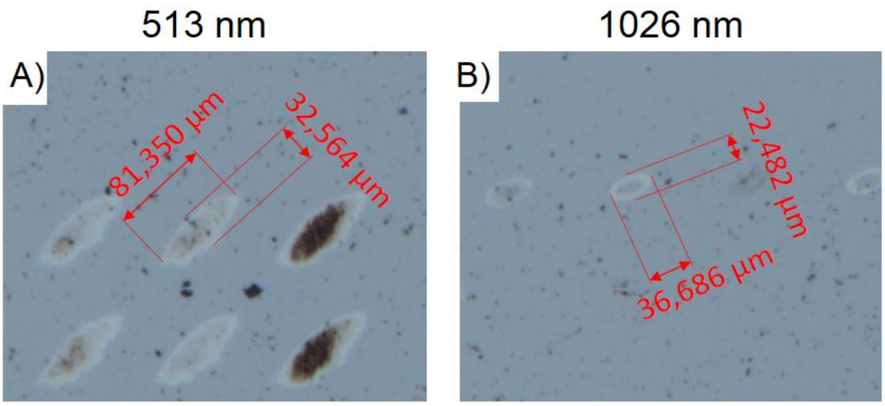

Figure S 11 Laser-induced damage on the silicon coverslip produced with different wavelengths. A) Single shots per spot with an average pulse energy of 30 µJ were used to produce the craters with the second harmonic wavelength. B) Single shots per spot with an average pulse energy of 20 µJ were used to produce the craters with the fundamental wavelength.

A)

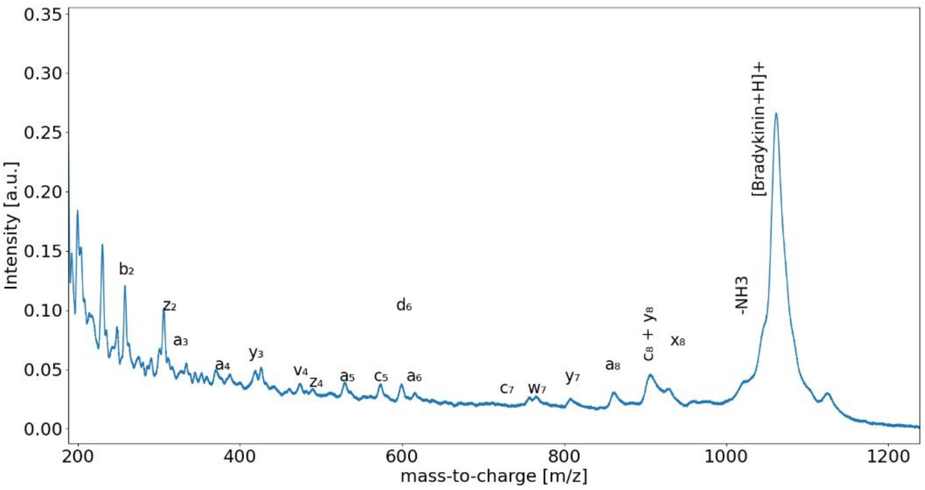

B)

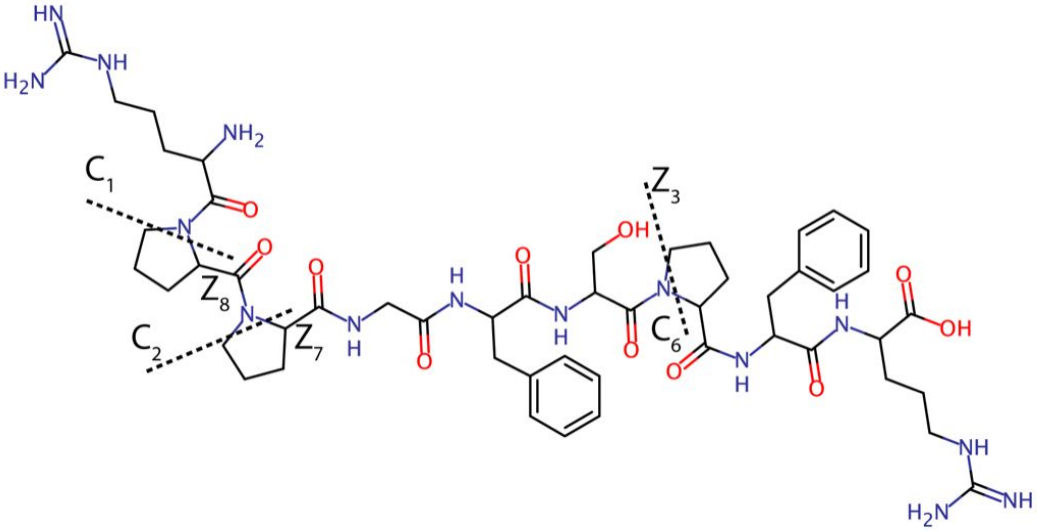

Figure S 12 Femtosecond mass spectrometry produces a vibrant pattern of bradykinin fragments. A) Mass spectrum of 100 µM bradykinin on silicon. B) Dotted lines indicate a possible Proline cleavage for the creation of c_n and z_n ions. An interesting observation was made with the amino acid Proline. Its position in bradykinin is at second, third, and seventh place. Fragment ions of the series c_n and z_n were missing at these positions. Since these fragments evolve through the cleavage between secondary nitrogen and alpha-carbon, the amino acid's structure must be considered. Proline possesses a five-member ring, which is included in the peptide backbone. The absence of fragment peaks suggests the unlikeliness of the double-cleavage event. For the same reasoning, z_3, z_7, and z_8 C-terminal fragments were not detected.

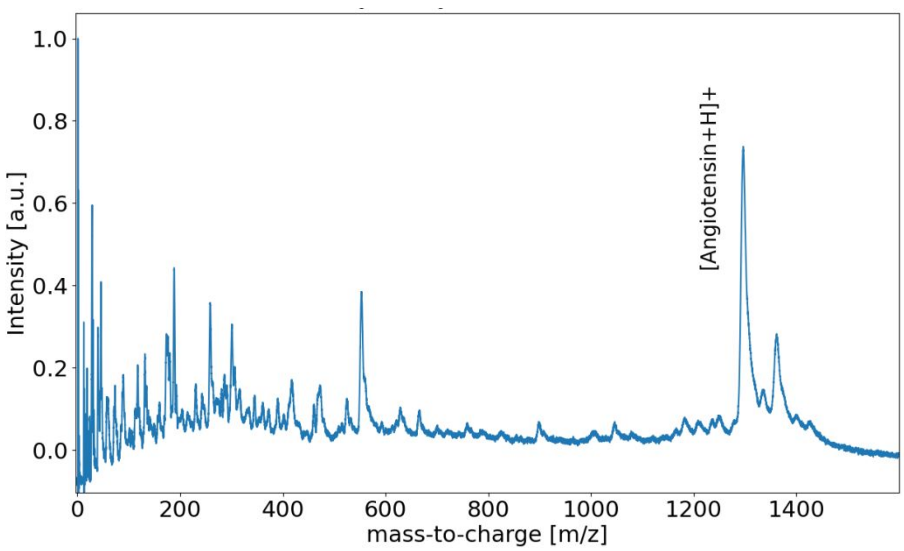

Figure S 13 Angiotensin mass spectrum produced with femtosecond pulses. The wavelength was 1026 nm and the specimen concentration 100 µM. In the mass range between 0 and 300 m/z substation ion signals are dominating. Smaller peaks between 300 and 1300 m/z are associated with angiotensin fragments.

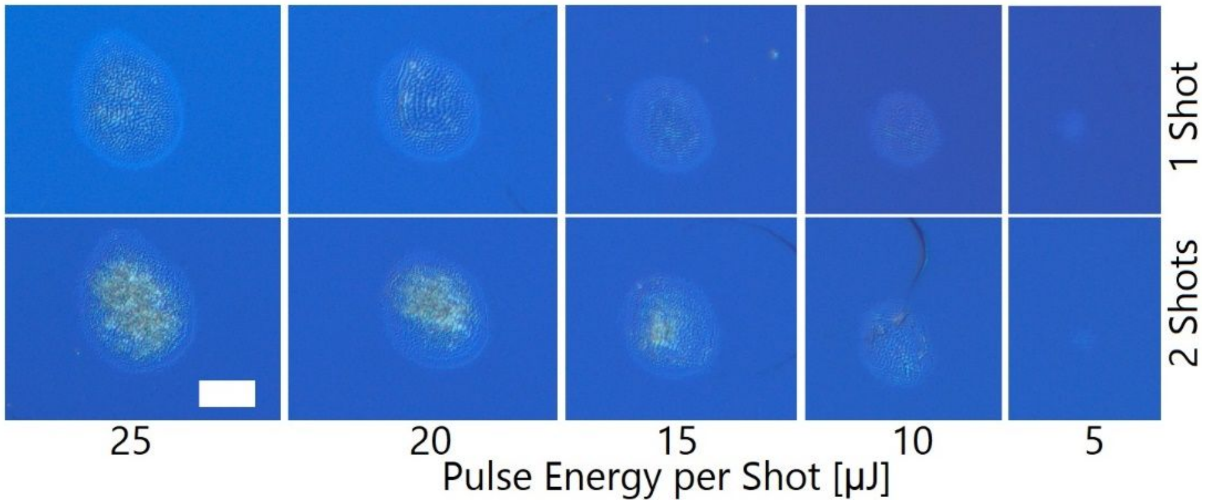

Figure S 14 Brightfield images of craters on a silicon wafer produced with femtosecond pulses. The irradiation wavelength was 1026 nm. The craters on the top row of the image were produced by a single shot on the same spot, while in the bottom row the craters were produced by a double shot. The experiment was conducted during ice sublimation to mimic the experimental conditions. The white bar indicates 100 µm.

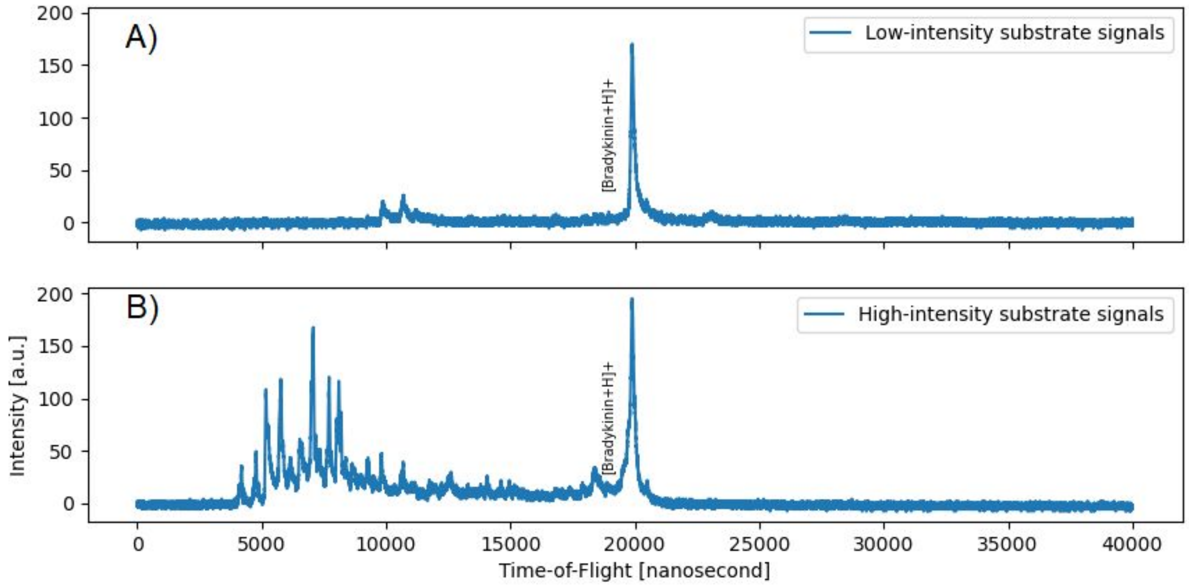

Figure S 15 Single shot mass spectra of 100 µM bradykinin from the same data set. A) Bradykinin arrives at the detector almost as a single species. B) Bradykinin signal is accompanied by substrate ions.

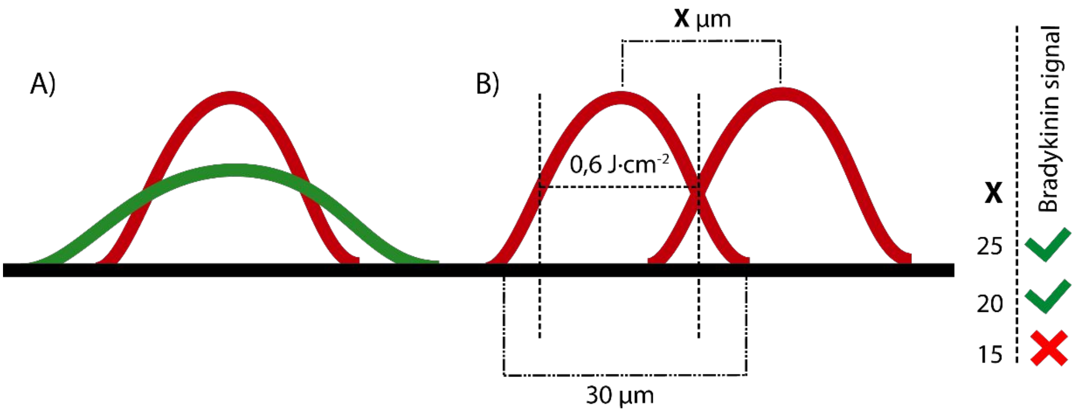

Figure S 16 Laser profile of the second harmonic (513 nm) and fundamental (1026 nm) during ablation. A) A comparison between 513 nm (green) and 1026 nm (red). B) Bradykinin signal onset dependence on the distance of adjacent pulses.

A) B)

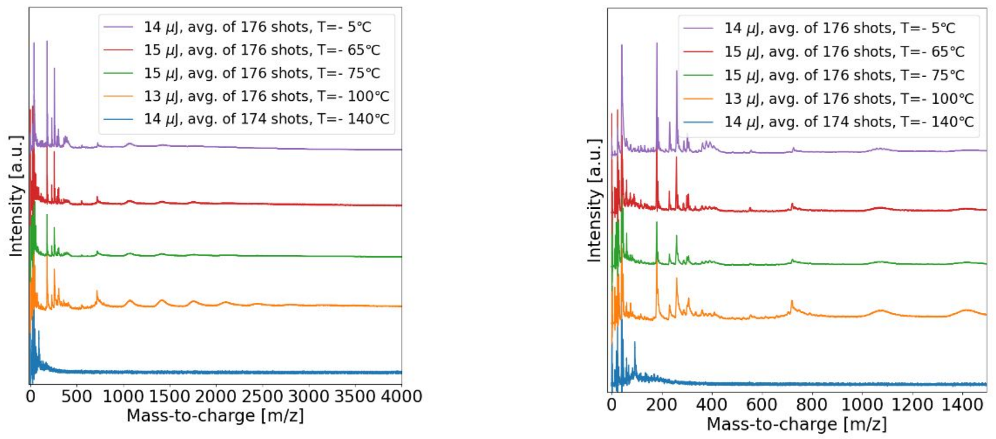

Figure S 17 Temperature-dependent mass spectra of fullerene C60 obtained with 1026 nm irradiation. The analyte concentration was 1 mM, and the pulse energies were between 13 – 15 µJ. A) A full range mass spectrum. B) A zoom-in of the original mass spectrum in the range between 700 – 2700 m/z.

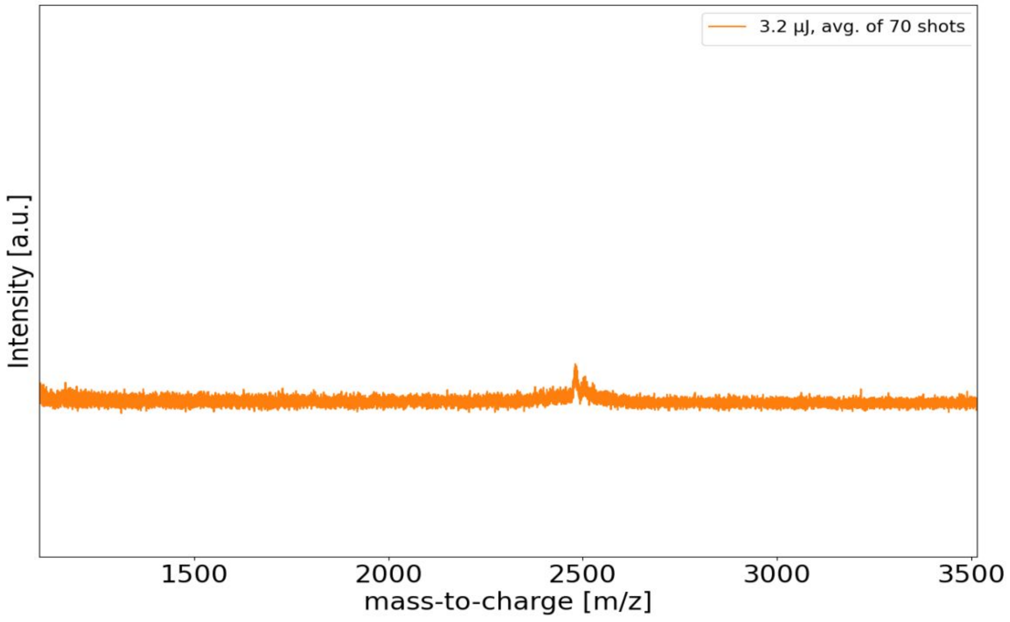

Figure S 18 Mass limit detection. Endothelin (2,491 kDa, 100 µM) is the largest detected molecule in the present work.

Table S 1 Fragmentation ions from bradykinin observed on different substrates. The masses presented in this table include a proton attachment. Indium tin oxide and chalcogenide glass are abbreviated as ITO and CG, respectively. Fragment ion identification was accomplished by comparison of theoretical and measured masses. Mass differences less than 2 Da were considered a match.

Fragment name	Average mass with a proton	Ions obtained with 513 nm on silicon	Ions obtained with 1026 nm on silicon	Ions obtained with 1026 nm on ITO	Ions obtained with 1026 nm on CG
b2	255,32	-	256,84	-	255,08
z2	306,11	305,79	306,24	-	-
a3	324,42	325,84	325,62	323,86	324,79
c3	367,46	368,80	369,40	368,01	-
y3	417,48	417,97	417,55	418,09	-
c4	424,5	425,61	425,68	-	-
v4	473,53	473,83	473,20	472,06	-
z4	490,56	490,06	490,09	-	-
a5	528,66	529,21	528,86	528,00	-
c5	571,68	572,65	572,63	571,95	-
d6	597,72	598,43	598,89	598,32	-
a6	615,74	614,66	615,78	614,40	613,31
a7 + y6	712,85 and 709,81	-	-	712,77	-
c7	755,88	756,44	756,47		-
w7	765,86	765,04	765,85	-	-
y7	806,93	805,62	806,50	-	-
a8	860,03	860,51	860,90	859,59	-
c8 + y8	903,06 and 904,04	903,48	905,30	904,54	-
x8	932,05	930,69	930,31	929,01	902,01
$-R_{Arginin}$	961,08	959,81	-	-	-
$-R_{Benzyl}$	970,10	971,75	-	-	-
-COOH	1016,21	1015,67	-	1014,40	-
-NH3	1044,2	1046,70	1045,37	-	-
Bradykinin+H+	1061,23	1060,91	1061,00	1060,84	1060,99
Bradykinin+K+	1100,33	1099,21	-	-	-
